# Cross-institutional automated multilabel segmentation for acute intracerebral hemorrhage, intraventricular hemorrhage, and perihematomal edema on CT

**DOI:** 10.1093/radadv/umaf012

**Published:** 2025-03-21

**Authors:** Jawed Nawabi, Georg Lukas Baumgaertner, Sophia Schulze-Weddige, Andrea Dell’Orco, Andrea Morotti, Federico Mazzacane, Helge Kniep, Frieder Schlunk, Maik Franz Hermann Boehmer, Burak Han Akkurt, Tobias Orth, Jana-Sofie Weissflog, Maik Schumann, Peter B Sporns, Michael Scheel, Uta Hanning, Jens Fiehler, Tobias Penzkofer

**Affiliations:** Department of Neuroradiology, Charité—Universitätsmedizin Berlin, Humboldt-Universität zu Berlin, Freie Universität Berlin, Berlin Institute of Health, Berlin 10117, Germany; Department of Radiology, Charité—Universitätsmedizin Berlin, Campus Virchow Klinikum, Humboldt-Universität zu Berlin, Freie Universität Berlin, Berlin Institute of Health, Berlin 13351, Germany; Department of Radiology, Charité—Universitätsmedizin Berlin, Campus Virchow Klinikum, Humboldt-Universität zu Berlin, Freie Universität Berlin, Berlin Institute of Health, Berlin 13351, Germany; Department of Neuroradiology, Charité—Universitätsmedizin Berlin, Humboldt-Universität zu Berlin, Freie Universität Berlin, Berlin Institute of Health, Berlin 10117, Germany; Department of Clinical and Experimental Sciences, Neurology Clinic, University of Brescia, Brescia 25123, Italy; Department of Brain and Behavioral Sciences, University of Pavia, Pavia 271000, Italy; U.C. Malattie Cerebrovascolari e Stroke Unit, IRCCS Fondazione Mondino, Pavia 271000, Italy; Department of Neuroradiology, University Medical Center Hamburg-Eppendorf, Hamburg 20251, Germany; Department of Neuroradiology, University Medical Center Freiburg, Freiburg 79106, Germany; Department of Radiology, University Hospital Muenster, Muenster 48149, Germany; Department of Radiology, University Hospital Muenster, Muenster 48149, Germany; Department of Radiology, Charité—Universitätsmedizin Berlin, Campus Virchow Klinikum, Humboldt-Universität zu Berlin, Freie Universität Berlin, Berlin Institute of Health, Berlin 13351, Germany; Department of Neuroradiology, Charité—Universitätsmedizin Berlin, Humboldt-Universität zu Berlin, Freie Universität Berlin, Berlin Institute of Health, Berlin 10117, Germany; Department of Radiology, Charité—Universitätsmedizin Berlin, Campus Virchow Klinikum, Humboldt-Universität zu Berlin, Freie Universität Berlin, Berlin Institute of Health, Berlin 13351, Germany; Department of Neuroradiology, University Medical Center Hamburg-Eppendorf, Hamburg 20251, Germany; Department of Neuroradiology, University Hospital Basel, Basel 4031, Switzerland; Department of Radiology, Neuroradiology and Nuclear Medicine, Stadtspital Zürich, Zürich 8063, Switzerland; Department of Neuroradiology, Charité—Universitätsmedizin Berlin, Humboldt-Universität zu Berlin, Freie Universität Berlin, Berlin Institute of Health, Berlin 10117, Germany; Department of Neuroradiology, University Medical Center Hamburg-Eppendorf, Hamburg 20251, Germany; Department of Neuroradiology, University Medical Center Hamburg-Eppendorf, Hamburg 20251, Germany; Department of Radiology, Charité—Universitätsmedizin Berlin, Campus Virchow Klinikum, Humboldt-Universität zu Berlin, Freie Universität Berlin, Berlin Institute of Health, Berlin 13351, Germany

**Keywords:** artificial intelligence, deep learning, segmentation, hemorrhage, intracerebral hemorrhage

## Abstract

**Background:**

Precise volume quantification of intracerebral hemorrhage (ICH), intraventricular hemorrhage (IVH), and perihematomal edema (PHE) is a critical parameter for guiding therapy decisions, monitoring therapeutic effects over time, and predicting patient outcomes.

**Purpose:**

To evaluate a nnU-Net-based deep learning model for automated, multilesion segmentation on non-contrast CT.

**Materials and Methods:**

Retrospective data from acute spontaneous ICH patients admitted to 4 stroke centers (2015-2022) and controls (2022-2023) were analyzed. Manual segmentations served as ground truth with repeated segmentations as reference standard. nnU-Net was trained (*n* = 775) using 5-fold cross-validation and tested on a holdout set (*n* = 189). Lesion detection, segmentation, and volumetric accuracy were evaluated using the Dice similarity coefficient (DSC) and Pearson correlation coefficients (r), with subanalyses for anatomical location and impact of other hemorrhage types (subarachnoid, subdural, or epidural hematoma). The model was validated on internal (*n* = 121) and external (*n* = 169) datasets. Processing time was compared to manual segmentation.

**Results:**

Test set sensitivity was 99% for ICH and PHE and 97% for IVH. Segmentation achieved a DSC of 0.91 (ICH), 0.71 (PHE), and 0.76 (IVH), with *r* = 0.99 (ICH, IVH) and *r* = 0.92 (PHE). DSC for lobar and deep hemorrhages were 0.90 and 0.92, respectively, and 0.70 in the brainstem, with other hemorrhage types showing no significant impact on segmentation accuracy, *P* > .05. For internal validation, DSC was 0.88 (ICH), 0.66 (PHE), and 0.80 (IVH), with r of 0.98, 0.88, and 0.98, respectively. External validation yielded DSC values of 0.85 (ICH), 0.61 (PHE), and 0.80 (IVH), with *r* values of 0.97, 0.85, and 0.96. Mean processing time was 18.2 s (±5 SD), compared to 18.01 min (±20.47 SD) for manual segmentations.

**Conclusion:**

nnU-Net enables reliable, time-efficient segmentation of ICH, PHE, and IVH, validated across multicenter, multivendor datasets of spontaneous ICH, showing potential to enhance clinical workflows.

**Abbreviations**

DICOM = Digital Imaging and Communications in Medicine; EDH = epidural hemorrhage; FDA = Food and Drug Administration; GPUs = graphics processing units; GT = ground truth; ICH = intracerebral hemorrhage; IVH = intraventricular hemorrhage; NA = not applicable; NCCT = non-contrast Computed Tomography; PHE = perihematomal edema; PACS = Picture Archiving and Communication System; RIS = radiology information systems; RVD = relative volume difference; SD = standard deviation; SAH = subarachnoid hemorrhage; SDH = subdural hemorrhage.

**Summary**

Our study validated the model’s ability to detect and segment intracerebral hemorrhage, intraventricular hemorrhage, and perihematomal edema on non-contrast CT scans, providing accurate, reproducible volume assessments for potential clinical application.

**Key Results**

- Our nnU-Net model achieved robust detection accuracies (97%-99% sensitivity) for intracerebral hemorrhage (ICH), perihematomal edema (PHE), and intraventricular hemorrhage (IVH).
- Segmentation performance was strong, with Dice scores of 0.91 (ICH), 0.71 (PHE), and 0.76 (IVH), supported by Pearson correlation coefficient of 0.99 (ICH, IVH) and 0.92 (PHE).
- Trained on multicenter, multivendor datasets with internal and external validation, the model demonstrated strong generalizability and clinical potential.

## Introduction

Intracerebral hemorrhage (ICH), along with perihematomal edema (PHE) and intraventricular hemorrhage (IVH), presents substantial clinical challenges due to its high morbidity and mortality rates.^1–3^ Accurate imaging assessments are pivotal, as their volumes directly impact therapy decisions and outcome predictions. ICH volume is crucial for predicting hematoma expansion, a potentially reversible therapeutic target, with the highest risk occurring within the first 3 h and being closely associated with neurological deterioration.^4^ Each 1 mL expansion in hematoma volume within the first 24 h correlates with a 5% higher risk of death or disability.^5^ Timely and accurate volume estimation is therefore essential to identify at-risk patients and guide early therapeutic interventions, such as blood pressure lowering or anticoagulation reversal.^5^^,^^6^ Surgical intervention is guided by ICH volume, which serves as a key criterion in minimally invasive clot removal trials for lobar or basal ganglia hemorrhages (30-80 mL) and deep ICH cases (30-100 mL).^7^^,^^8^ Longitudinal volume tracking is essential for evaluating these therapies and monitoring progression, where reduced PHE serves as a surrogate marker.^2^^,^^7^^,^^9^ Meanwhile, residual ICH volume often serves as a secondary outcome in surgical trials, further emphasizing the need for precise quantification.^7^ Prognostic value varies significantly by ICH location; smaller thalamic volumes (<15 mL) predict worse outcomes compared to larger lobar hemorrhages.^10^ Reliable quantification of PHE and IVH volumes is equally critical—guiding timely interventions like external ventricular drainage or thrombolysis and assessing therapies aimed at hematoma clearance with PHE reduction.^3^^,^^11^ Current workflows rely on manual segmentation or the simplified ABC/2 method, which often overestimates ICH volume and lacks precision,^12^ potentially leading to unnecessary interventions or delayed care.^9^ Inaccurate measurements of PHE similarly pose risks by either delaying critical treatments or prompting overly aggressive interventions, increasing associated risks. Automated tools promise standardized, accurate volume assessments essential for improving therapy decisions and outcome predictions.^13^ However, previous studies addressing all 3 lesion types have been limited in scope and generalizability.^14^^,^^15^ Further, current commercial tools primarily focus on hemorrhage detection alone and lack the capability to simultaneously segment ICH, IVH, and PHE, underscoring the need for a more advanced approach. We present a multicenter-trained and validated nnU-Net model for automated detection and segmentation of ICH, PHE, and IVH in spontaneous ICH, facilitating therapy decisions, longitudinal lesion tracking, and outcome prognostication in acute care.

## Materials and methods

### Study design

This retrospective multicenter study received approval from the ethics committees of Site 1 (protocol number EA1/035/20), Site 2 (protocol number WF-054/19), Site 3 [2017-233-f-S], and Site 4 (protocol number 20190099462). All protocols and procedures adhered to the Declaration of Helsinki. Given the study’s retrospective nature, patient consent was not required. All data processing, including model training and inference, was handled on local servers.

### Data collection and patient demographics

**Figure 1.**
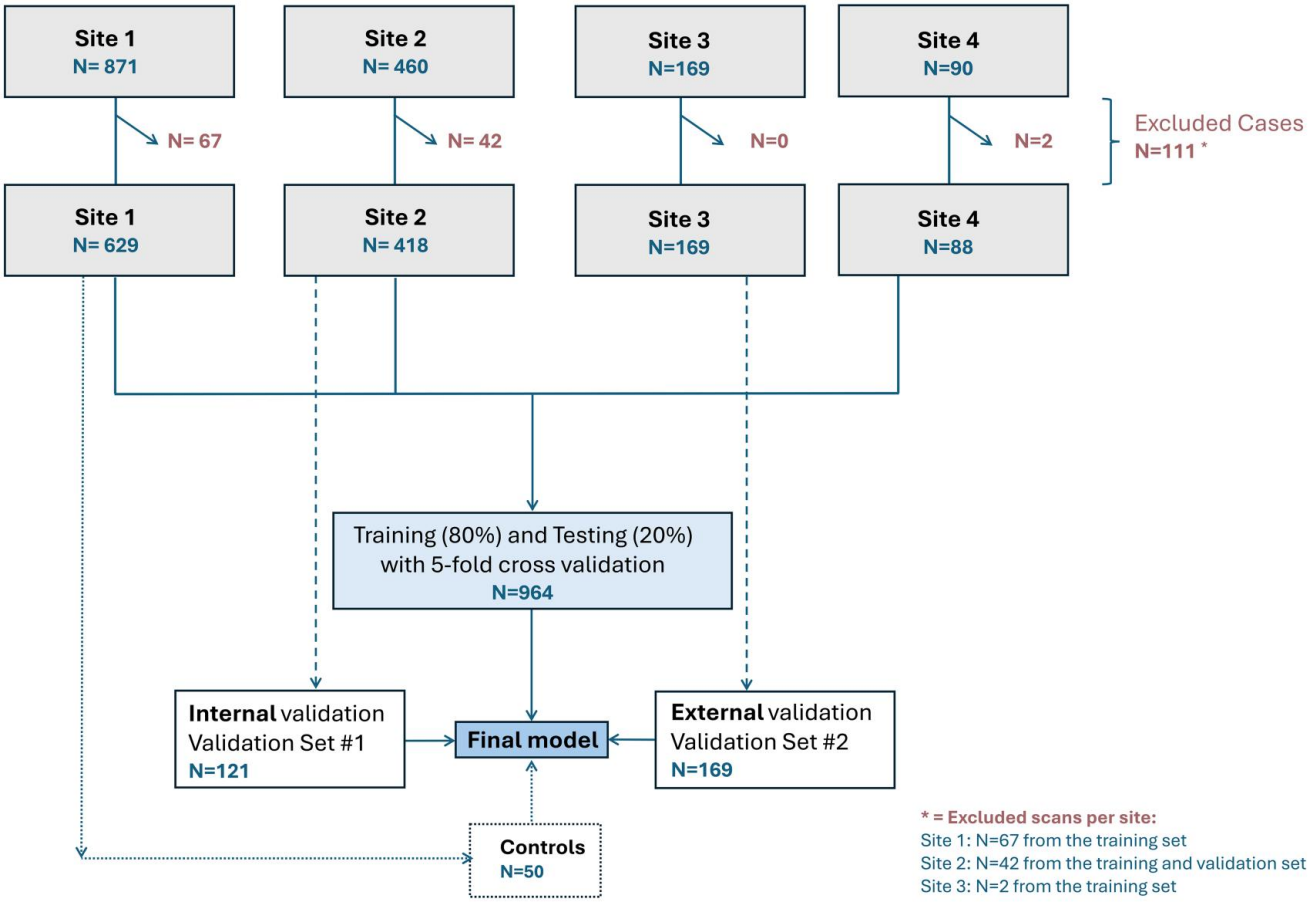
Study design and data flow for model training and validation. The figure outlines the patient distribution and data flow from 4 study sites for the training, testing, and validation of the deep learning model. Data were collected from 4 sites. Data from Site 1, Site 2, Site 4, and an additional set of healthy controls were used for model training and testing (total *n* = 964) using a 5-fold cross-validation approach (80% training, 20% testing). Internal validation (Validation Set 2, *n* = 121) was performed using a separate subset of Site 2 data. Data from Site 3 (*n* = 169) were reserved for external validation (Validation Set 2). Exclusion of images was due to insufficient quality of segmentations (*n* = 111).

Retrospective analysis included patients from 4 stroke centers between 2015 and 2022 (Site 1: 2015-2020; Site 2: 2015-2020; Site 3: 2015-2017; Site 4: 2019-2022). Patients met the following criteria: (1) primary, spontaneous ICH; (2) age ≥18 years; and (3) baseline non-contrast Computed Tomography (NCCT) within 36 h of onset or last known well status. Exclusion criteria were (1) non-primary ICH (eg, trauma, vascular malformation, tumor, or ischemic stroke), (2) non-parenchymal bleeding, and (3) insufficient imaging data. Control NCCT scans from individuals without imaging pathologies (aged ≥18 years) were obtained from Site 1 (2022-2023). The training and validation datasets were supplemented with previously reported studies meeting these criteria, focusing on imaging predictors of ICH outcomes and IVH growth^16^^,^^17^ (Supplementary Material), with the data distribution shown in Figure 1.

### Image acquisitions

NCCT scans followed site-specific protocols using 14 scanner models from 4 manufacturers (Tables S1 and S2).

### Image quality assessment and reference standard

Imaging analysis protocol and quality assessment procedures are detailed in the Supplementary Material. Qualitative analyses of CT scans assessed the presence of IVH, subarachnoid hemorrhage (SAH) extension, subdural hemorrhage (SDH) or epidural hemorrhage (EDH), and hemorrhage location. Quantitative analysis involved detailed manual segmentation of ICH, PHE, and IVH regions, referred to as the ground truth, which was performed once, except for repeated segmentations used to establish the reference standard. The reference standard consisted of a subset of *N* = 30 scans from Validation Set 2, with repeated manual segmentation performed by a trained reader, T.O. (radiology resident with 5 years of experience), and reviewed by an expert, J.N. (board-certified neuroradiologist with 8 years of experience). Discrepancies were resolved through consensus.^18^

### Deep learning network architecture

We utilized the adaptable nnU-Net framework for semantic segmentation of biomedical images, characterized by its dynamic adjustment to datasets and a detailed U-Net-like architecture (Figure S1), with specifics on its encoder-decoder structure and parameter groups detailed in the Supplementary Material.^19^^,^^20^

### Experimental protocol

#### Training and testing

Training involved subjects from Site 1, Site 2, and Site 4, with 80% of the data used for model training and validation. This 80% was split using a 5-fold cross-validation approach, where each fold contributed to training and validation to boost model robustness. Final predictions were made by combining all folds. Models were trained for 1000 epochs using an initial learning rate of 0.01, batch size of 12, and a combined loss function of Dice loss and categorical cross-entropy for multi-class segmentation (Supplementary Material).^21^ Loss optimization employed stochastic gradient descent.^22^ Training was conducted using Python (v3.8.10)^23^ and nnU-Net (v2.0)^24^ on NVIDIA graphics processing units (GPUs), including Quadro RTX 8000, A100, and H100.^25^ The code used for training and inference is available from the original GitHub repository.^24^ Due to data protection policies, the neural network weights cannot be shared, but collaborations can be considered upon regulatory approval. The remaining 20% of the data were kept separate for testing purposes, ensuring the evaluation was performed on unseen data.

#### Validation

Our segmentation network’s robustness was tested using 2 datasets: the internal validation set (Validation Set 1) from site 2 and the external validation set (Validation Set 2) from Site 3. Additional details are available in the Supplementary Material.

#### Analysis time

A comparative time efficiency analysis was performed between manual segmentations by T.O. and the network’s automated segmentation on the test and external validation cohorts. nnU-Net segmentation time was averaged across the external and internal validation sets to prevent data leakage and maximize data pool size, with all results reported as mean values ± standard deviation (SD).

#### Statistical analyses

For the descriptive analysis, the test and validation cohorts (Validation Set 1 and 2) were compared to the training cohort using independent samples *t*-tests. The model’s automated detection accuracy was assessed using standard evaluation metrics, including sensitivity, specificity, and overall accuracy. Volume estimation performance was measured using the Dice similarity coefficient (DSC), absolute volume error (AVE), relative volume difference (RVD), and Pearson correlation coefficients.^26–28^ Subgroup analyses focused on lesions >1 mL for clinical relevance, different anatomical locations, and cases with coexisting SAH, EDH, and SDH, evaluating accuracy with DSC and Pearson correlation coefficient.^29^ The confidence intervals (CIs) were calculated based on the mean and SD of the sample data using bootstrapping. A paired samples *t*-test compared performance metrics between the reference standard and network’s predictions. Methodological details, including formulas and subgroup analyses, are provided in the Supplementary Material. All statistical analyses were performed using the scikit-learn library (Python v3.8.10; Python Software Foundation).^23^^,^^30^

## Results

### Characteristics of the study cohort

The training and test cohorts showed comparable ICH volumes (median [IQR]: 22.95 mL [8.31-52.75] vs 21 mL [5.93-42.09]; *P*-value of .145), ensuring consistency for model validation (Table 1). However, significant differences were noted in IVH volumes, which were higher in the test cohort compared to the training cohort (median [IQR]: 8.31 mL [2.48-19.74] vs 7.98 mL [2.66-19.77]; *P* < .001), although the presence of IVH was not significantly different (47.6% vs 49%; *P*-value of .746). In contrast, Validation Sets 1 and 2 demonstrated both significantly higher IVH volumes (Validation 1: 12.1 mL [3.24-31.64]; *P* < .001; Validation 2: 12.49 mL [7.04-23.27]; *P* < .001) and IVH presence (Validation 1: 59%; *P* < .001; Validation 2: 59%; *P* < .001) compared to the training set. PHE volume was lower in Validation Cohort 1 (median [IQR]: 16.2 mL [6.63-34.97]; *P* = .003) and higher in Validation Cohort 3 (median [IQR]: 32.38 mL [13.89-60.13]; *P*-value of .002), while the training and test cohorts showed comparable PHE volumes (median [IQR]: 20.66 mL [9.36-42.8] vs 20.48 mL [8.01-41.54]; *P*-value of .315). Additional differences in clinical characteristics, including Glasgow Coma Scale score, symptom onset to imaging time, and ICH location, are outlined in Table S3.

**Table 1.** Patient and imaging characteristics across training, test, and validation sets.

Characteristics	Training set(*N* = 775)	Test set (*N* = 189)	Validation Set 1 (*N* = 121)	Validation Set 2 (*N* = 169)
Age [years], median (IQR)	73 (60-79)	73 (63-81)	69 (75-80)	69 (55-79)
Sex [female], *n* (%)	325 (41.9)	97 (51.3%)	45 (37.5)	92 (53.5)
Systolic blood pressure [mmHg], median (IQR)	170 (144.5-200)	163.50 (142-190)	159 (132-188)	196 (178.75-242.5)
History of hypertension, *n* (%)	606 (78.2)	156 (82.5)	80 (66.1)	92 (53.5)
Oral anticoagulation, *n* (%)	209 (27)	47 (24.9)	30 (24.8)	35 (20.3)
Antiplatelet medication, *n* (%)	355 (45.8)	92 (48.7%)	57 (47.1)	59 (34.4)
GCS at baseline, median (IQR)	13 (7-15)	12 (6.5; 15)	13 (6-15)	12 (6; 13)
Symptom onset to imaging [hours], median (IQR)	4.5 (1.81-15.85)	3.92 (1.48-11.04)	2.2 (1.28-4.74)	NA
mRS 4-6, *n* (%)	574 (73.7)	148 (78.3)	84 (69.4)	100 (58.1)
Missings, *n* (%)	20 (3.1)	1 (0.5)	1 (0.8)	4 (2.3)
ICH location				
Lobar, *n* (%)	342 (44.1)	80 (42.3)	56 (46.3)	75 (43.6)
Deep, *n* (%)	313 (40.4)	80 (42.3)	54 (44.6)	85 (49.4)
Brainstem/pons, *n* (%)	40 (5.2)	9 (4.8)	1 (0.8)	5 (2.9)
Cerebellum, *n* (%)	80 (10.3)	20 (10.6)	10 (8.3)	7 (4.1)
ICH Vol [mL], median (IQR)	22.95 (8.31-52.75)	21 (5.93-42.09)	24.53 (7.8-56.46)	28.57 (11.14-55.39)
IVH presence, *n* (%)	380 (49)	90 (47.6)	59 (49.17)	59 (35.12)
IVH Vol [mL], median (IQR)	7.98 (2.66-19.77)	8.31 (2.48-19.74)	12.1 (3.24-31.64)	12.49 (7.04-23.27)
PHE Vol [mL], median (IQR)	20.66 (9.36-42.8)	20.48 (8.01-41.54)	16.2 (6.63-34.97)	32.38 (13.89-60.13)

The volumes reported for ICH, PHE, and IVH are derived from ground truth segmentations.

Abbreviations: GCS = Glasgow Coma Scale; NA = not applicable; ICH = intracerebral hemorrhage; IVH = intraventricular hemorrhage; mRS = modified Rankin Scale; PHE = perihematomal edema; SD = standard deviation; Vol = volume.

### Performance of lesion segmentation, detection, and volume quantification

**Figure 2.**
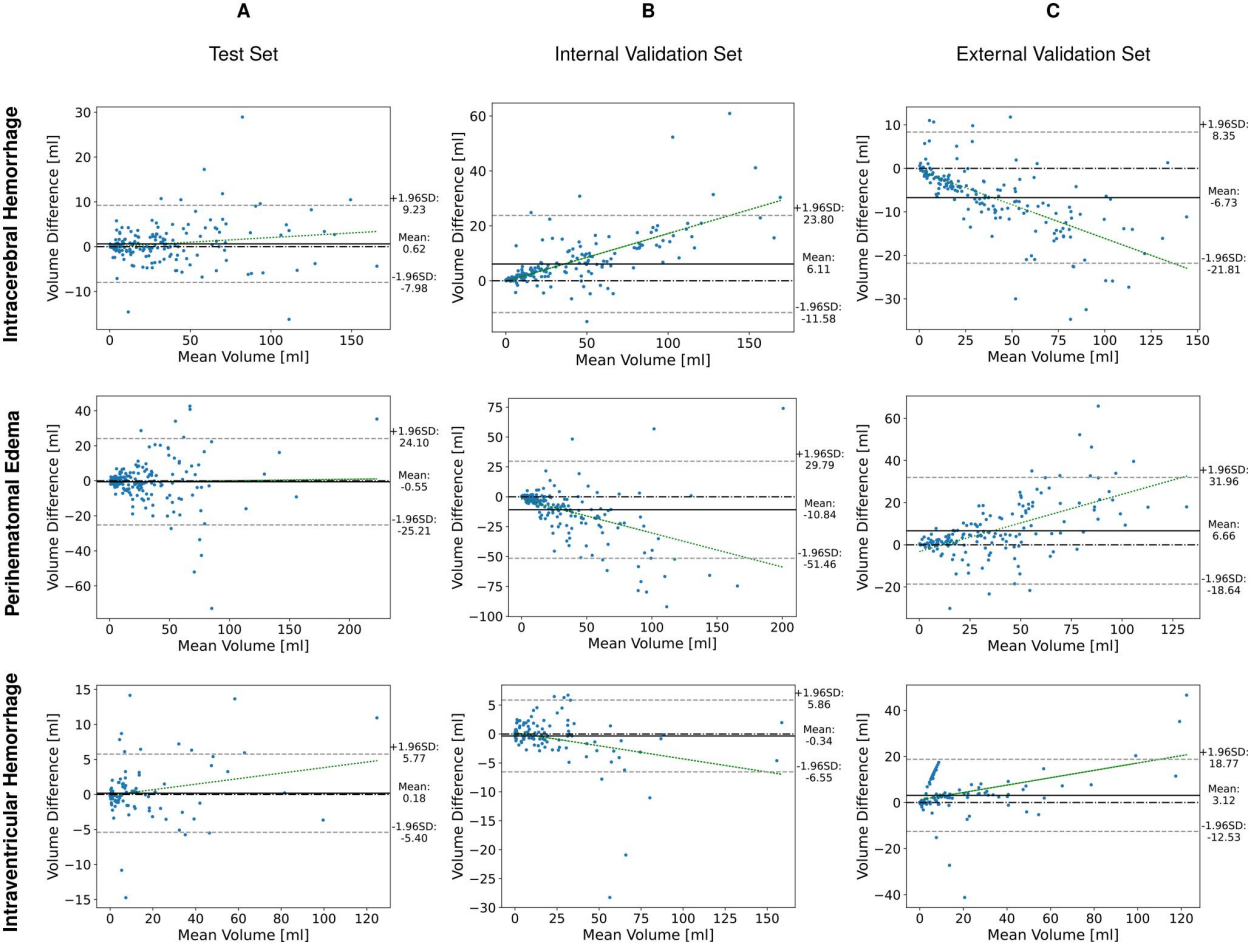
Bland-Altman analysis for performance on test set versus validation sets. Bland-Altman Analysis for assessing the concordance between automated segmentation volumes and the established ground truth for intracerebral hemorrhage (ICH; upper row), perihematomal edema (PHE; mid row), and intraventricular hemorrhage (IVH; bottom row). The *x*-axis represents the mean volume derived from automated segmentation, while the *y*-axis depicts the volume difference. Where positive values indicate underestimation by the automated method relative to the manual gold standard, whereas negative values suggest overestimation. The central solid line denotes the mean absolute volume deviation from the true volume, with the surrounding dotted lines representing the bounds of 1 SD. Panel A illustrates the analysis within the test set, Panels B and C demonstrate the findings in validation sets (internal and external sets, respectively).

Volume analysis is supported by the Bland-Altman analysis (Figure 2) and volume correlation plots (Figure S3). In both figures, the first row corresponds to ICH, the middle row to PHE, and the last row to IVH. Columns represent the test set (A), internal validation set (B), and external validation set (C).

#### Intracerebral hemorrhage

**Figure 3.**
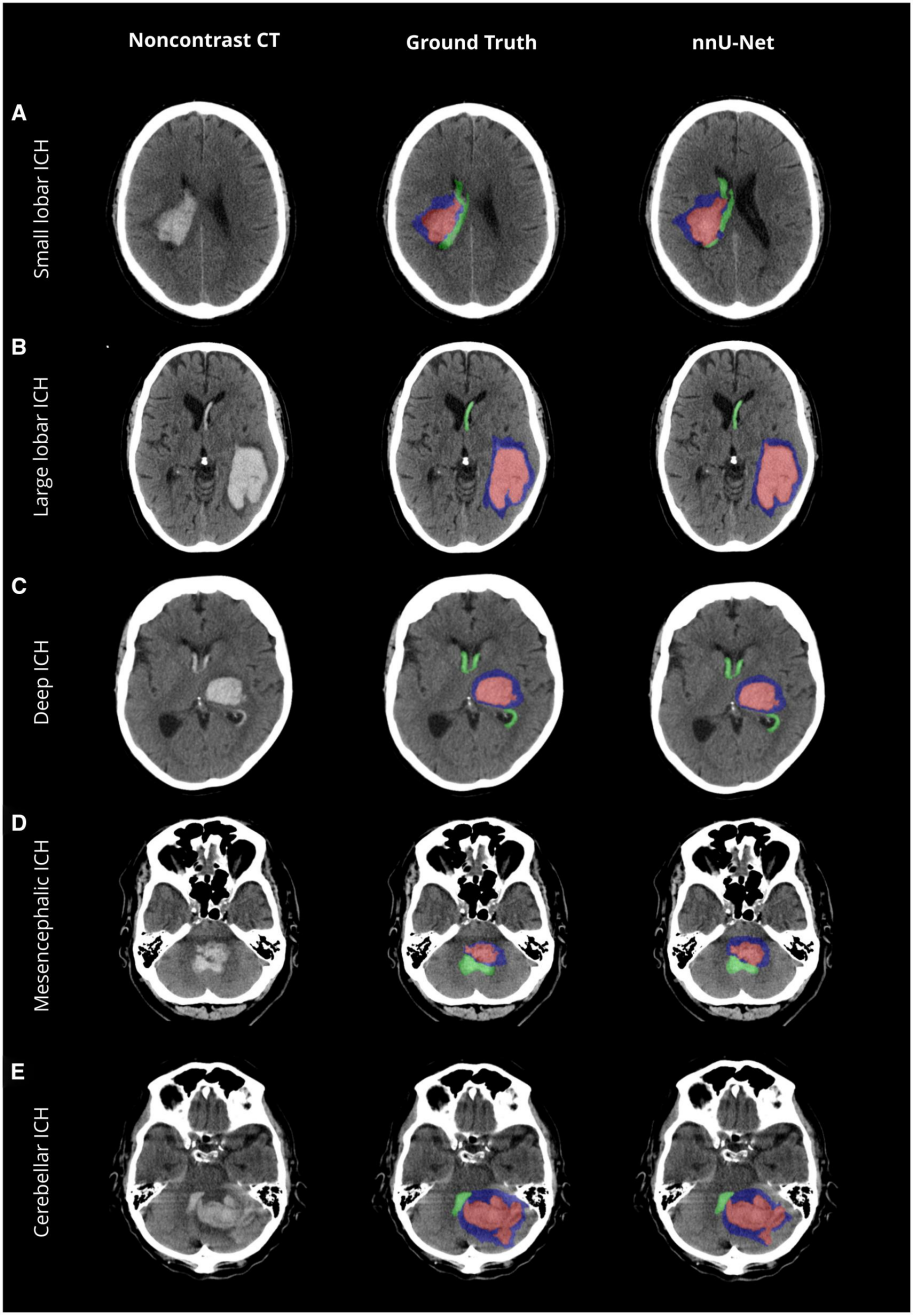
Comparative visualization of model predictions versus ground truth across various anatomical locations. Non-contrast CT scans with the original data (left column), ground truth segmentations (mid column), and the network’s predicted segmentations (nnU-Net, right column) for different anatomical contexts. Each row represents a distinct anatomical context, illustrating the model’s ability to identify and segment key pathological features. The color-coding is as follows: intracerebral hemorrhage (ICH) is highlighted in red; intraventricular hemorrhage extension (IVH) is marked in green; and perihematomal edema (PHE) is depicted in blue.

**Figure 4.**
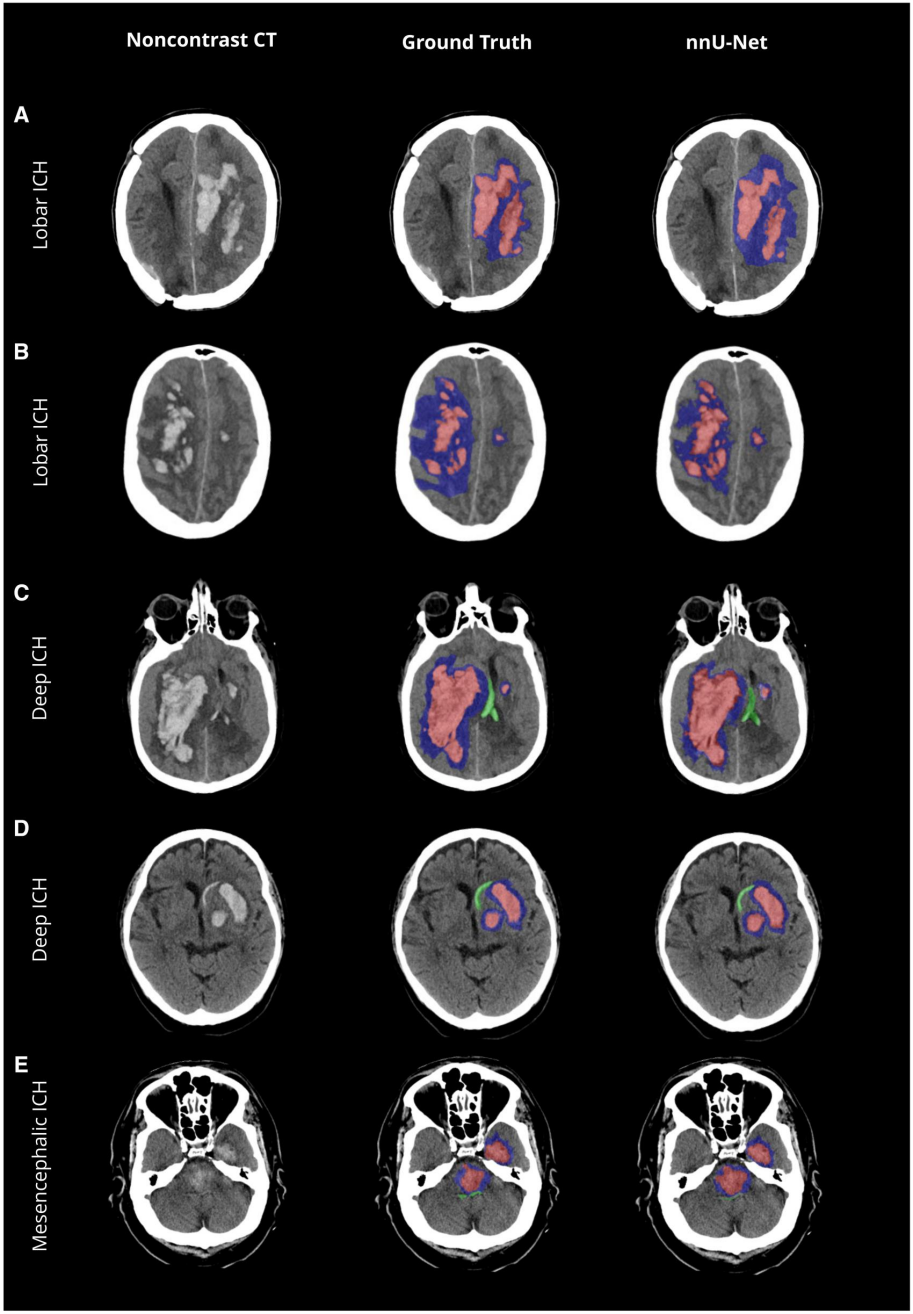
Comparative visualization of model predictions versus ground truth across heterogeneous hematoma configurations. Non-contrast CT scans with the original data (left column), ground truth segmentations (mid column), and the network’s predicted segmentations (nnU-Net, right column) for different anatomical contexts. Each row represents a distinct anatomical context, illustrating the model’s ability to identify and segment key pathological features. The color-coding is as follows: intracerebral hemorrhage (ICH) is highlighted in red; intraventricular hemorrhage extension (IVH) is marked in green; and perihematomal edema (PHE) is depicted in blue.

**Figure 5.**
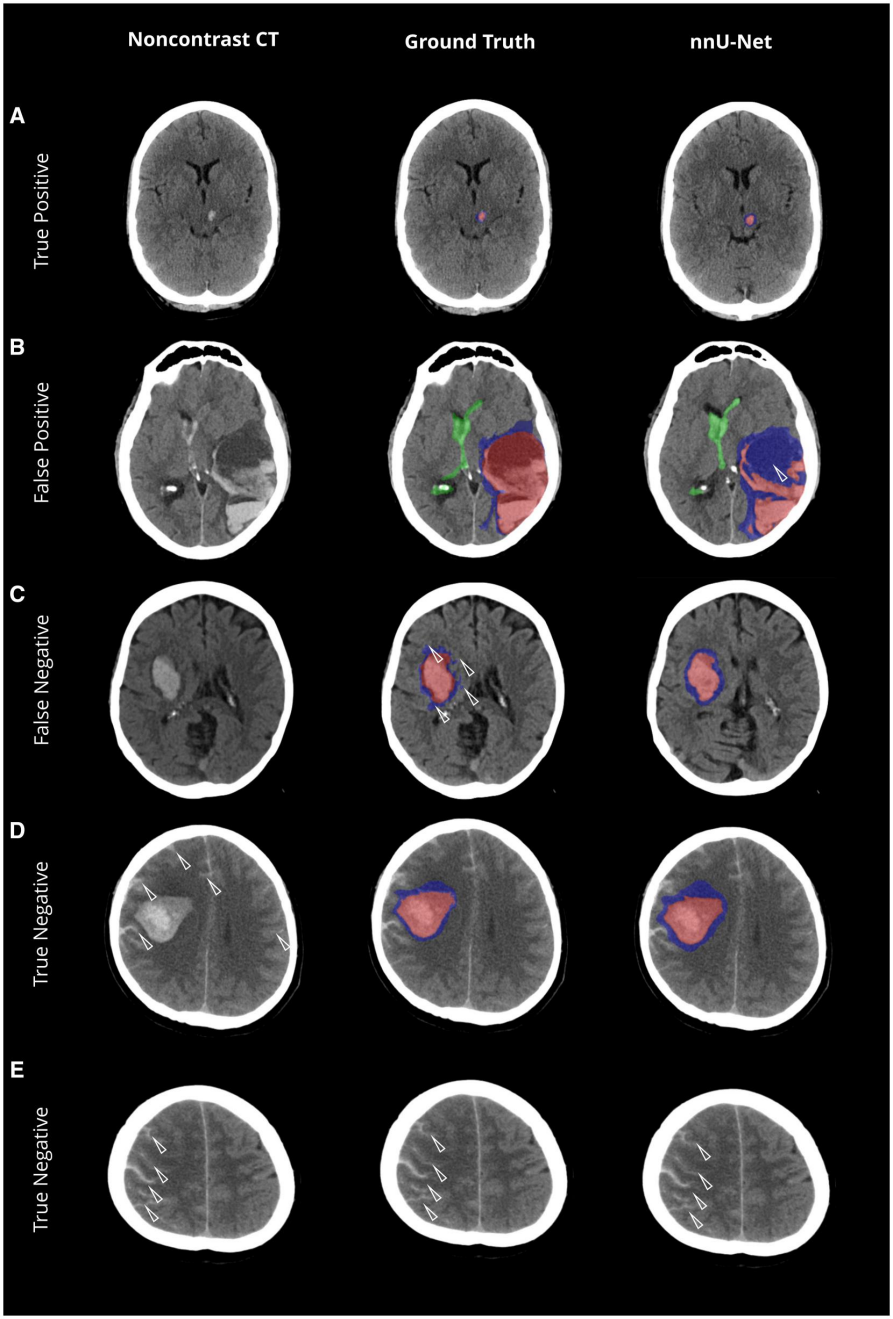
Evaluation of intracerebral and perihematomal edema predictions—a visual comparison of model accuracy and misclassifications. Non-contrast CT scans with the original data (left column), ground truth segmentations (mid column) and the network’s predicted segmentations (nnU-Net, right column). (A) High accuracy in identifying small intracerebral hemorrhage (ICH; <5 mL). (B) Misclassification error in identifying a hypodense portion of the hemorrhage as perihematomal edema (PHE) potentially attributed to the similar densities as the latter as indicated by the white arrow. (C) Correct classification of PHE; however, showing that the network tended to smooth contours relative to the ground truth. (D-E) High accuracy in not capturing subarachnoid hemorrhage extension (SAH & E; white arrows) along the cortical fissures as ICH. The color-coding is as follows: ICH is highlighted in red; and PHE is depicted in blue.

The network achieved a median DSC of 0.91 (±0.01) for ICH against the reference standard, with a *P*-value of .12 (Table 2). Illustrative cases highlight the network’s ability to handle both regular (Figure 3) and complex and irregular (Figure 4) as well as small ICH (Figure 5A), achieving a high detection accuracy and sensitivity of 99% (±1%). The volume difference was minimal at 5% (±10%), with an absolute difference of −0.62 mL (±0.65), indicating close alignment with the reference standard. Bland-Altman analysis (Figure 2A) revealed a nearly tripled average volume difference of −1.69 mL for ICH volumes ≥30 mL. Importantly, the absolute deviation increased for larger volumes, while the relative deviation decreased from an average of 6% for smaller volumes to 3% for larger volumes. Additionally, a Pearson correlation coefficient of 0.99 confirmed robust agreement in volume estimation, as supported in the volume correlation plots.

**Table 2.** Evaluation of nnU-Net framework: lesion segmentation, volume estimation, and detection accuracy across the test dataset in comparison with the ground truth.

Lesion type	Performance metrics	Test dataset	Reference standard
ICH metrics	Mean DICE score	0.87 ± 0.02	0.83 ± 0.05
	Median DICE score	0.91 ± 0.01	0.86 ± 0.03
	*t*-Test vs test dataset; *P*-value		0.1962
	Volume difference (%)	5% ± 10%	7% ± 19%
	Absolute difference (mL)	−0.63 ± 0.65	0.76 ± 1.64
	Pearson correlation coefficient GT vs AI	0.99	0.98
	Detection accuracy (%)	99% ± 1%	100% ± 0%
	Detection sensitivity (%)	99% ± 1%	100% ± 0%
	Detection specificity (%)	NA	NA
PHE metrics	Mean DICE score	0.67 ± 0.02	0.67 ± 0.07
	Median DICE score	0.71 ± 0.02	0.71 ± 0.04
	*t*-Test vs test dataset; *P*-value		0.7521
	Volume difference (%)	12% ± 9%	−10% ± 13%
	Absolute difference (mL)	0.57 ± 1.84	−4.32 ± 5.42
	Pearson correlation coefficient GT vs AI	0.92	0.91
	Detection accuracy (%)	98% ± 2%	100% ± 0%
	Detection sensitivity (%)	99% ± 1%	100% ± 0%
	Detection specificity (%)	NA	NA
IVH metrics	Mean DICE score	0.51 ± 0.07	0.82 ± 0.10
	Median DICE score	0.68 ± 0.09	0.84 ± 0.04
	*t*-Test vs test dataset; *P*-value		0.001014
	Volume difference (%)	−1% ± 10%	11% ± 4%
	Absolute difference (mL)	−0.87 ± 0.83	−2.10 ± 1.8
	Pearson correlation coefficient GT vs AI	0.99	1
	Detection accuracy (%)	82% ± 6%	100% ± 0%
	Detection sensitivity (%)	97% ± 4%	100% ± 0%
	Detection specificity (%)	70% ± 9%	100% ± 0%
IVH threshold metrics (>1 mL)	Mean DICE score	0.65 ± 0.06	0.82 ± 0.10
	Median DICE score	0.76 ± 0.05	0.84 ± 0.04
	*t*-Test vs test dataset; *P*-value		0.068174
	Detection accuracy (%)	90% ± 5%	100% ± 0%
	Detection sensitivity (%)	86% ± 8%	100% ± 0%
	Detection specificity (%)	93% ± 5%	100% ± 0%

Performance metrics (1) for segmentation evaluation, including Dice score analysis, represented through mean and median values, along with pairwise comparison among various datasets; (2) volume estimation accuracy, utilizing both relative and absolute volume discrepancies, complemented by a Pearson correlation coefficient assessment between actual GT volumes and those predicted by the AI; and (3) segmentation metrics for IVH exceeding 1 mL delineated in a separate subsection. Metrics are reported as mean ± 95% CI unless stated otherwise.

Abbreviations: AI = artificial intelligence; ICH = intracerebral hemorrhage; IVH = intraventricular hemorrhage; DICE = Dice Similarity Coefficient; GT = ground truth; NA = not applicable; PHE = perihematomal edema.

#### Perihematomal edema

For PHE, the median DSC was 0.71 (±0.02), with a *P*-value of .75 (Table 2). Misclassifications occurred in cases with density overlap to hyperacute ICH or alterations to adjacent white matter due to microangiopathy (Figure 5B-D). The volume difference was 12% (±9%), with an absolute difference of 0.57 mL (±1.84). Bland-Altman analysis showed limits of agreement ranging from −25 mL to +24 mL. Despite this, a Pearson correlation coefficient of 0.92 indicated strong agreement in volume estimation, supported by scatterplot analysis. Detection accuracy remained high at 98%, with sensitivity reaching 99%.

#### Intraventricular hemorrhage

**Figure 6.**
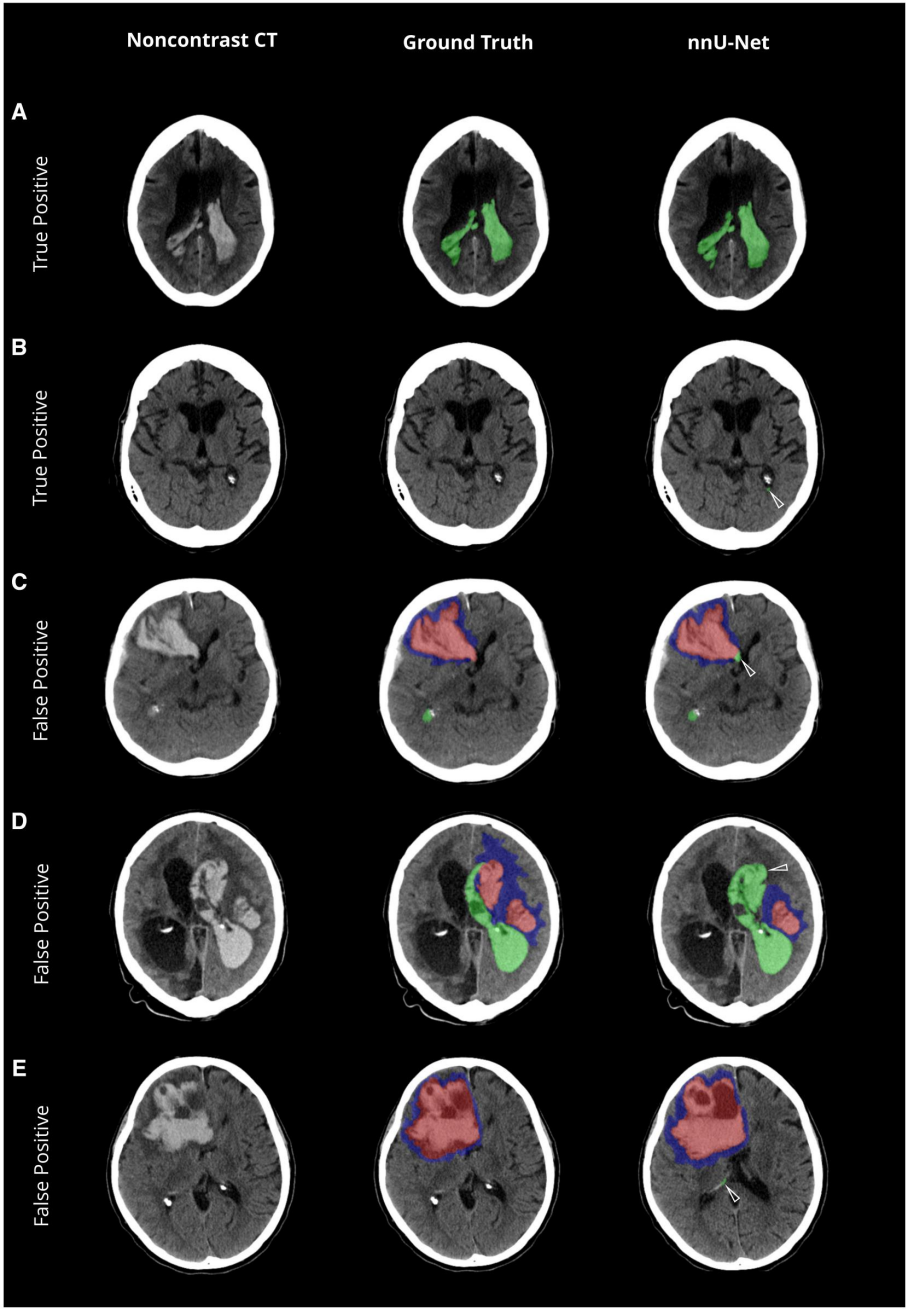
Evaluation of intraventricular hemorrhage predictions—a visual comparison of model accuracy and misclassifications. Non-contrast CT scans with the original data (left column), ground truth segmentations (mid column), and the network’s predicted segmentations (nnU-Net, right column). (A) High accuracy in the prediction of a large and heterogenous intraventricular hemorrhage (IVH) extension into the lateral ventricles. (B) Correct classification of a small IVH in the left lateral ventricle juxtaposed to the missed classification in the ground truth. (C-D) Misclassification of intracerebral hemorrhage (ICH) as IVH as indicated by the white arrow in case of overlapping anatomical context and IVH-like shape. (E) Misclassification of IVH as indicated by white arrow in case of IVH-shaped formation of the choroid plexus due to ICH-induced mass effect. The color-coding is as follows: ICH is highlighted in red; IVH extension is marked in green; and perihematomal edema is depicted in blue.

IVH segmentation showed a median DSC of 0.68 (±0.09), slightly below ICH and PHE (Table 2 and Figure 6A). Sensitivities and specificities remained robust down to IVH volumes of 0.2 mL (Table S4, Figure 6B, and Figure S4). Median DSC even improved for IVH volumes above 1 mL (0.76 ± 0.05). Misclassifications occurred predominantly in cases with anatomical overlap or morphological similarities, such as a compressed choroid plexus caused by ICH-induced mass effect resembling thin IVH (Figure 6C-E). Despite these challenges, volume quantification demonstrated high accuracy, with a volume difference of −1% (±10%) and an absolute difference of −0.87 mL (±0.83). Pearson Correlation Coefficient remained high at 0.99. Bland-Altman plots showed limits of agreement ranging from −5 mL to +6 mL for IVH.

#### Performance across anatomical locations

Performance by anatomical location demonstrated high precision for ICH and PHE. For ICH, median DSCs were 0.90 ± 0.01 (lobar) and 0.92 ± 0.01 (deep), with Pearson correlation coefficients of 0.95 and 0.99 (Table 3). PHE achieved median DSCs of 0.71 ± 0.02 (lobar) and 0.72 ± 0.02 (deep), with correlations of 0.90 and 0.93. For IVH, median DSCs were 0.71 ± 0.07 (lobar) and 0.82 ± 0.04 (deep), with correlations of 0.98 and 0.95. However, IVH segmentation was more challenging in the brainstem, where median DSC fell to 0.33 ± 0.21. Despite this, volume correlations for IVH remained strong across most areas with a mean volume difference of −1% and −3% for lobar and deep and −24% and 26% for brainstem and cerebellum, supported by illustrative cases in Figures 5 and 6.

**Table 3.** Location-specific performance evaluation: segmentation and volume estimation metrics across different locations in the test dataset.

Lesion type	Performance metrics	Lobar	Deep	Brainstem	Cerebellum
		*N* = 80	*N* = 80	*N* = 9	*N* = 20
ICH metrics	Mean DICE score	0.87 ± 0.03	0.88 ± 0.03	0.65 ± 0.17	0.82 ± 0.06
	Median DICE score	0.90 ± 0.01	0.92 ± 0.01	0.70 ± 0.29	0.87 ± 0.05
	*t*-Test vs lobar; *P*-value	—	0.78762	<0.0001	0.10145
	Volume difference (%)	−1% ± 8%	−2% ± 3%	104% ± 161%	17% ± 31%
	Absolute difference (mL)	−1.23 ± 2.71	−0.55 ± 0.71	1.75 ± 3.57	−0.26 ± 1.13
	Pearson correlation coefficient GT vs AI	0.95	0.99	0.76	0.98
	Detection accuracy (%)	99% ± 2%	99% ± 2%	100% ± 0%	100% ± 0%
	Detection sensitivity (%)	99% ± 2%	100% ± 0%	100% ± 0%	100% ± 0%
	Detection specificity (%)	NA	0% ± 0%	NA	NA
PHE metrics	Mean DICE score	0.69 ± 0.03	0.68 ± 0.03	0.49 ± 0.12	0.61 ± 0.07
	Median DICE score	0.71 ± 0.02	0.72 ± 0.02	0.50 ± 0.18	0.65 ± 0.08
	*t*-Test vs lobar; *P*-value	—	0.86315	0.00014	0.03346
	Volume difference (%)	12% ± 10%	1% ± 8%	88% ± 143%	22% ± 22%
	Absolute difference (mL)	2.75 ± 3.71	−1.64 ± 2.22	1.82 ± 2.96	0.74 ± 2.43
	Pearson correlation coefficient GT vs AI	0.9	0.93	0.8	0.94
	Detection accuracy (%)	99% ± 3%	98% ± 3%	100% ± 0%	95% ± 10%
	Detection sensitivity (%)	99% ± 3%	100% ± 0%	100% ± 0%	100% ± 0%
	Detection specificity (%)	NA	0% ± 0%	NA	0% ± 0%
IVH metrics	Mean DICE score	0.46 ± 0.11	0.61 ± 0.10	0.26 ± 0.21	0.39 ± 0.19
	Median DICE score	0.62 ± 0.2	0.79 ± 0.07	0.22 ± 0.29	0.33 ± 0.49
	Dice score *t*-test vs lobar *P*-value	—	0.045641	0.23261	0.45215
	Volume difference (%)	−1% ± 17%	−3% ± 10%	−24% ± 82%	26% ± 46%
	Absolute difference (mL)	−2.53 ± 4.73	−0.91 ± 1.26	−5.53 ± 5.89	−0.30 ± 1.82
	Pearson correlation coefficient GT vs AI	0.98	0.99	0.45	0.95
	Detection accuracy (%)	81% ± 9%	86% ± 8%	88% ± 22%	65% ± 22%
	Detection sensitivity (%)	96% ± 7%	98% ± 5%	100% ± 0%	100% ± 0%
	Detection specificity (%)	72% ± 13%	74% ± 15%	79% ± 36%	36% ± 30%
IVH threshold metrics (>1 mL)	Mean DICE score	0.62 ± 0.09	0.72 ± 0.08	0.33 ± 0.21	0.56 ± 0.19
	Median DICE score	0.71 ± 0.07	0.82 ± 0.04	0.32 ± 0.29	0.65 ± 0.28
	Dice score *t*-test vs lobar *P*-value	—	0.02072112	0.13046	0.6709
	Detection accuracy (%)	92% ± 6%	91% ± 6%	89% ± 22%	80% ± 19%
	Detection sensitivity (%)	90% ± 11%	88% ± 10%	75% ± 43%	78% ± 28%
	Detection specificity (%)	94% ± 7%	95% ± 7%	100% ± 0%	82% ± 24%

Performance metrics (1) for segmentation evaluation, including Dice score analysis, represented through mean and median values, along with pairwise comparison among various datasets; (2) volume estimation accuracy, utilizing both relative and absolute volume discrepancies, complemented by a Pearson Correlation Coefficient assessment between actual GT volumes and those predicted by the AI; and (3) segmentation metrics for IVH exceeding 1 mL delineated in a separate subsection. Metrics are reported as mean ± 95% CI unless stated otherwise.

Abbreviations: AI = artificial intelligence; ICH = intracerebral hemorrhage; IVH = intraventricular hemorrhage; DICE = Dice Similarity Coefficient; GT= ground truth; NA = not applicable; PHE = perihematomal edema.

#### Performance across extraaxial hemorrhage types

As shown in Figure S5, the presence of extraaxial hemorrhages did not significantly affect ICH segmentation accuracy. For cases with SDHs/EDHs, the mean DSC was 0.87 ± 0.05 compared to 0.87 ± 0.14 without (*P* = .72, Table S5). Similarly, for SAHs, the mean Dice score was 0.90 ± 0.04 with SAHs and 0.86 ± 0.14 without (*P* = 0.31), as illustrated in Figure 5E.

### Performance across different validation sets

The network’s segmentation performance was consistent across validation sets (Table 4), with ICH median DSCs of 0.88 (±0.01) in Set 1, 0.85 (±0.01) in Set 2, and 0.90 (±0.01) in Set 3, comparable to the test set (0.91 ± 0.01). In Validation Set 1, the limits widened to −12 mL to +24 mL in the Bland-Altman analysis, while in Validation Set 2, the limits were narrower, ranging from −22 mL to +8 mL. For PHE, median DSCs ranged from 0.61 to 0.67 across validation sets, aligning closely with the test set (0.67 ± 0.03). Bland-Altman analysis revealed limits of agreement for Validation Set 1 widening to −51 mL to +30 mL, while in Validation Set 2, the limits were slightly narrower, at −19 mL to +32 mL. IVH segmentation showed more variability, with median DSCs of 0.80 (±0.03 and ±0.08) for Sets 2 and 3, slightly exceeding Set 1 and the test set (0.67 ± 0.13 and 0.76 ± 0.05, respectively). A visual comparison of segmentation accuracy across the test set, validation set, and the reference standard is presented in Figure S6. Pearson correlation coefficient (0.97-0.99 for ICH, >0.90 for PHE and IVH) confirmed strong volume estimation across all validation sets. Bland-Altman analysis for Validation Set 1 showed limits of agreement ranging from −12 mL to +6 mL for IVH, indicating greater variability for smaller volumes (<10 mL). In Validation Set 2, the limits of agreement expanded further, ranging from −19 mL to +13 mL, reflecting increased variability for smaller IVH volumes.

**Table 4.** Evaluation of nnU-Net framework: lesion segmentation, volume estimation, and detection accuracy across validation sets.

Lesion type	Performance metrics	Validation Set 1	Validation Set 2
ICH metrics	Mean DICE score	0.82 ± 0.03	0.81 ± 0.02
	Median DICE score	0.88 ± 0.01	0.85 ± 0.01
	*t*-Test vs test dataset; *P*-value	0.0056844	0.00025
	Volume difference (%)	−18% ± 3%	21% ± 6%
	Absolute difference (mL)	−6.11 ± 1.25	6.84 ± 1.41
	Pearson correlation coefficient GT vs AI	0.99	0.97
	Detection accuracy (%)	97% ± 2%	99% ± 2%
	Detection sensitivity (%)	97% ± 3%	99% ± 2%
	Detection specificity (%)	NA	NA
PHE metrics	Mean DICE score	0.61 ± 0.02	0.57 ± 0.02
	Median DICE score	0.65 ± 0.01	0.61 ± 0.02
	*t*-Test vs test dataset; *P*-value	<0.001	<0.001
	Volume difference (%)	55% ± 21%	−11% ± 5%
	Absolute difference (mL)	10.78 ± 2.86	−6.65 ± 2.04
	Pearson correlation coefficient GT vs AI	0.86	0.93
	Detection accuracy (%)	97% ± 3%	98% ± 2%
	Detection sensitivity (%)	97% ± 3%	98% ± 2%
	Detection specificity (%)	NA	NA
IVH metrics	Mean DICE score	0.61 ± 0.06	0.46 ± 0.06
	Median DICE score	0.78 ± 0.04	0.62 ± 0.18
	*t*-Test vs test dataset; *P*-value	0.0210991	0.34598
	Volume difference (%)	−1% ± 10%	−10% ± 10%
	Absolute difference (mL)	−0.87 ± 0.83	0.19 ± 1.44
	Pearson correlation coefficient GT vs AI	0.99	0.91
	Detection accuracy (%)	89% ± 4%	78% ± 6%
	Detection sensitivity (%)	94% ± 4%	79% ± 8%
	Detection specificity (%)	83% ± 8%	76% ± 11%
IVH threshold metrics (>1 mL)	Mean DICE score	0.49 ± 0.07	0.68 ± 0.05
	Median DICE score	0.67 ± 0.13	0.80 ± 0.03
	*t*-Test vs test dataset; *P*-value	0.72394	0.001785
	Detection accuracy (%)	89% ± 4%	72% ± 7%
	Detection sensitivity (%)	81% ± 7%	63% ± 9%
	Detection specificity (%)	99% ± 2%	90% ± 8%

Performance metrics (1) for segmentation evaluation, including Dice score analysis, represented through mean and median values, along with pairwise comparison among various datasets; (2) volume estimation accuracy, utilizing both relative and absolute volume discrepancies, complemented by a Pearson correlation coefficient assessment between actual ground truth (GT) volumes and those predicted by the AI; and (3) segmentation metrics for IVH exceeding 1 mL delineated in a separate subsection. Metrics are reported as mean ± 95% CI unless stated otherwise.

Abbreviations: AI = artificial intelligence; ICH = intracerebral hemorrhage; IVH = intraventricular hemorrhage; DICE = Dice Similarity Coefficient; GT= ground truth; NA = not applicable; PHE = perihematomal edema.

### Analysis time

The trained model processed cases in an average of 18.2 s (±5 SD), significantly faster than manual segmentation (18.01 min ±20.47 SD). Processing times were 20.67 s (±5 SD) for the internal validation set and 15.3 s (±5 SD) for the external validation set.

## Discussion

This study presents an automated deep learning model based on nnU-Net for precise detection and segmentation of ICH, PHE, and IVH using multicenter and multivendor datasets. The model achieved robust performance in lesion detection and volume estimation. Test set median DSCs were 0.91 for ICH, 0.71 for PHE, and 0.68 for IVH, with validation datasets showing similar results (ICH: 0.85-0.90, PHE: 0.61-0.67, IVH: 0.76-0.80 for lesions >1 mL). Bland-Altman analysis confirmed minimal volume deviations (−0.63 mL for ICH, 0.57 mL for PHE, and −0.87 mL for IVH), with limits of agreement smallest for IVH (−5 to 6 mL) and largest for PHE (−25 to 24 mL). Extraaxial hemorrhages (eg, SAH, SDH) showed no significant impact on ICH segmentation accuracy (*P* = .72). High detection accuracy was achieved across lesions (99% for ICH, 98% for PHE, and 82% for IVH), with sensitivity and specificity exceeding 90% for IVH volumes as small as 0.2 mL. Segmentation times were reduced to 18.2 s, significantly faster than manual segmentation (18 min).

The network demonstrated high segmentation precision for ICH, maintaining excellent DSC in challenging cases with irregular shapes or multifocal bleeding. Minor volume deviations were observed in cases exceeding 100 mL, which is consistent with findings reported by Sharrock et al. in their 3D U-Net “DeepBleed,”^31^ which highlights areas for refinement. Hyperacute bleeding, which can mimic the hypodense radiological phenotype of PHE due to adjacent hypodense regions, further emphasizes this need. Despite having the lowest DSC, PHE segmentation aligned with the reference standard, reinforcing reliability and mirroring results from previous studies.^18^ Additionally, the high Pearson correlation coefficient underscored the network’s effectiveness in volumetric quantification, a clinically relevant measure surpassing voxel-based contour replication. Sensitivity and specificity remained above 90% for IVH volumes even as small as 0.2 mL. However, segmentation proved challenging with smaller volumes. The DSC’s sensitivity to minor voxel misclassifications disproportionately affected small-scale IVH, a well-documented limitation.^32^ To improve specificity, we applied a threshold for lesions above 1 mL, consistent with Food and Drug Administration (FDA) standards for intracranial hyperdensities.^29^ Variability in IVH volumes across validation cohorts also likely contributed to observed differences in DSC.

The network’s performance remained stable across independent validation sets, including datasets from previously unseen CT manufacturers in Validation Set 2, demonstrating robust generalizability. Despite challenges from imaging artifacts, the network consistently delivered reliable results across anatomically complex regions (Figure S7). Additionally, the presence of other intracranial hemorrhages did not compromise the model’s accuracy in segmenting ICH, further underscoring its robustness.

Implementing the nnU-Net model in clinical radiology for CT scans poses several challenges, including the need for high-performance GPUs, which may limit accessibility in some settings. Integrating with radiology information systems (RIS) and Picture Archiving and Communication System (PACS), ensuring Digital Imaging and Communications in Medicine (DICOM) compatibility, and training radiologists to interpret and refine AI-assisted results are essential steps.^33^ Workflow adjustments, along with continuous validation and monitoring, will be critical to maintain accuracy tailored to individual requirements and diverse imaging systems.^34^

While our retrospective design may introduce bias, the multicenter cohort and 2 validation sets bolster the robustness of our findings. Limitations include potential segmentation errors and the exclusion of key patient populations, such as those with traumatic ICH, which restricts the model’s generalizability to broader clinical scenarios. Lower DSC for brainstem ICH likely reflects case rarity; targeted strategies like oversampling or augmentation could mitigate imbalances and further enhance performance.

We present a robust nnU-Net model for automated detection and segmentation of ICH, PHE, and IVH across multicenter datasets, facilitating therapy decisions, longitudinal tracking, and outcome prognostication in acute care. Expanding the dataset to include underrepresented anatomical regions and diverse ICH etiologies will be key to enhancing generalizability and clinical utility. Prospective validation is essential to confirm its applicability in real-world acute care settings.

## Supplementary Material

umaf012_Supplementary_Data

## Data Availability

The datasets that support the findings of our study are available upon reasonable request from the corresponding author. All data requests will first be reviewed by the institutional data security department/board, and based on their decision, access to the datasets may be granted. The configuration details of the nnU-Net used in this study have been added to the article to ensure transparency and reproducibility. However, the neural network weights cannot be shared due to the data protection policies of our institute. These measures are in place to mitigate the risk of inadvertently encoding sensitive information within the weights. We are open to establishing collaborations with individual institutions upon reasonable request, contingent on obtaining the necessary regulatory approvals.
