# Supplementary material for: Cross-institutional automated multilabel segmentation for acute intracerebral hemorrhage, intraventricular hemorrhage, and perihematomal edema on CT
Source: Radiol Adv. 2025 Mar 21;2(2):umaf012. doi: 10.1093/radadv/umaf012 (PMC12429176; doi:10.1093/radadv/umaf012)
Supplement: umaf012_Supplementary_Data [file umaf012_Supplementary_Data.zip › Supplementary Material_No track changes.Editorial Office.revision titles.docx]

Supplementary Material

Data Collection and Patient Demographics

We included data previously reported in studies of on ICH outcome prediction (total n=520; site 1: n=54, site 2: n=298, and data from site 3: n=168, which were reserved for external validation and not used for model training)^1^. Additionally, data from a study on the prediction of IVH growth (n=291 patients) were included^2^, with overlapping data from the latter reported study^1^: site 1 (n=54), site 2 (n=298), along with additional data from site 1 (n=725) and site 2 (n=418). Data from site 4 (n=88) were also added. Data from site 3 were fully excluded from this study, as IVH segmentations were not available at that time; hence, they were not included in the initial model training.

Image Quality Assessment and Reference Standard

Axial NCCT images were utilized, with slice thicknesses ranging from 1 mm to 5 mm, anonymized and stored in Digital Imaging and Communications in Medicine (DICOM) format. Quality assessments were conducted by A.M. and G.L.B. (biomedical informatics) through instance number and slice number checks, as well as filtering scans with few slices (A.M. and G.L.B., both biomedical informatics).^3^ Two-dimensional DICOM images were then converted to a three-dimensional structure using the Neuroimaging Informatics Technology Initiative format (NIfTI), re-orienting the images if necessary and resampling to a slice thickness of 5 mm. Additional subjective quality assessment was performed for the presence of artifacts, slice missing, and low axial resolution. CT scans were analyzed for the presence of IVH and hemorrhage location, followed by the imaging annotation for the three different labels ICH, PHE, and IVH as described previously.^4^ Volume regions of interest (VOI) were manually derived for each label using region segmentation on each slice with a polygon tool using the ITK Snap software (version 3.8.0).^5^ Ground truth segmentations for the training and validation datasets were performed once, with repeated segmentations conducted only for the creation of the reference standard to ensure robustness and reliability. Investigators from multiple centers contributed to the image segmentation, each with substantial experience in ICH imaging. The team included F.M. from Pavia, Italy (6 years of ICH imaging training; neurology resident); J.N., who contributed during tenures in both Hamburg, Germany (8 years of training; board-certified neuroradiologist), and later in Berlin, Germany (currently with 8 years of experience); U.H. from Hamburg, Germany (11 years of training; board-certified neuroradiologist); M.F.H.B. and B.H.A. from Münster, Germany (11 and 7 years of training; board-certified radiologist and radiology resident, respectively); and T.O. from Berlin, Germany (5 years of training; radiology resident). All residents were previously trained in segmentation techniques by J.N., who provided oversight and conducted quality control to ensure consistency and accuracy across centers. All investigators were trained blinded to subject identity, clinical information as well as the other readers’ interpretations. This segmentation methodology was validated by our previous study, which demonstrated outstanding interobserver reliability, as reflected by Intraclass Correlation Coefficients: 0.998 for ICH, 0.979 for IVH, and 0.886 for PHE.^4^ Notably, intrarater agreement was even higher, with coefficients between 0.98 to 0.99, underscoring the consistency and reproducibility of our segmentation process.^4^ All images used in the development and evaluation of the deep learning model were subjected to a central quality check: An expert in ICH imaging (J.N.) reviewed all images for IVH presence, evaluated the segmentation accuracy of the three labels, and made corrections where necessary. In the final step, we implemented a refined subjective assessment method by adopting a batched strategy, utilizing an HTML table layout, to display all scans in a web browser which were reviewed by an experienced investigator (J.N). Any scans that did not meet the necessary criteria were excluded, ultimately leading to the exclusion of a total of N=111 images after conducting both objective and subjective quality assessments as described.^3^

Deep Learning Network Architecture

Many different architectural designs exist to implement semantic segmentation. In this study, we employed the nnU-Net framework, a state-of-the art automated segmentation tool specifically engineered for biomedical image segmentation.^6^ The nnU-Net pipeline automates key steps such as data preprocessing, augmentation, and measures to prevent overfitting. These processes are embedded within the network's architecture and executed using a custom, published library (<https://github.com/MIC-DKFZ/batchgenerators>). Therefore, this method distinguishes itself by dynamically adjusting to each new dataset, thereby avoiding the need for static hyperparameters.^7^ Interrelated hyperparameters are grouped into three distinct groups: First, the blueprint parameters, which are held constant across all datasets; second, the inferred parameters, which are tailored dynamically to meet the unique demands of each dataset; and third, the empirical parameters, which cannot be predefined and must instead be derived from the data itself.^7^ Besides that, the nnU-Net network architecture mirrors that of the U-Net, employing an encoder-decoder structure enhanced with skip connections and an output stride of 1, as elaborated in Supplementary Figure 1. The encoder captures the essential contextual information necessary to distinguish among the three classes: ICH, PHE, and IVH. The decoder then progressively restores this information to the original image resolution, merging the upsampled contextual data from the preceding layers with the finer-resolution feature maps via skip connections.^7^ The network's depth, determined by the input size, encompassed seven stages of downsampling and upsampling.^6^ Architecturally, the network adopts a standard configuration with two blocks at each resolution level for both encoder and decoder. Each block comprises a sequence of convolution, instance normalization, and leaky ReLU nonlinearity, establishing a balance between complexity and performance.^8^ Our model used the following configuration:

| **Parameter** | **Value** |
| --- | --- |
| Model | 3d_fullres |
| Batch size | 2 |
| Patch size | 20 voxels x 320 voxels x 256 voxels |
| Spacing | 4 mm x 0.45 mm x 0.45 mm |
| Normalisation Sheme | CTNormalisation |
| Number of stages | 7 |
| Number of poolings per axis | 2, 6, 6 |
| Kernel sizes | 3 x (1 x 3 x 3), 4 x (3 x 3 x 3) |

The configuration details of the nnUNet used in this study have been added to the article to ensure transparency and reproducibility. However, the neural network weights cannot be shared due to the data protection policies of our institute. These measures are in place to mitigate the risk of inadvertently encoding sensitive information within the weights. We are open to establishing collaborations with individual institutions upon reasonable request, contingent on obtaining the necessary regulatory approvals.

Training and Testing

Subjects from Site 1; Site 2, Site 4 where combined for training and optimization of hyperparameters. We allocated 20% of the data for testing purposes. The remaining 80% of the dataset was used in a 5-fold cross-validation framework. In each fold, we divided this 80% into five equal parts. Four of these parts, constituting 64% of the total dataset, were used for training, while the fifth part was reserved for validation. This approach ensures that each segment of the dataset is used for both training and validation, enhancing the robustness of our model. For the final prediction, all five folds, each representing a separately trained instance of our nnU-Net model, were utilized collectively. It is important to note that the 20% of the dataset designated for testing was kept entirely separate and was not used in either the training or validation phases.

Models were trained for 1000 epochs with a learning rate of 0.01, and a batch size of 12 images. Dice loss complemented with the categorical cross-entropy loss were used as loss function.^9^ The categorial Cross-Entropy Loss is a Softmax activation plus a Cross-Entropy loss and considered as a popular loss function for multi-classification problems, which takes the output probabilities (P) and measures the distance from the truth values according to the following formula:

$$L_{CE}=-\sum_{i=1}^{C} w_{c}log\frac{\exp\left( x_{n,c} \right)}{\sum_{i=1}^{C} \exp\left( x_{n,i} \right)}y_{n,c} (Eq. 1)$$

where x is the input, y is the target, w is the weight, and C is the number of classes.

The Dice Similarity Coefficient (DSC) quantifies the volumetric overlap between the segmented outcome and the ground truth.^10^ It calculates this by comparing set A, which consists of the foreground voxels in the ground truth, with set B, the equivalent foreground voxels in the segmentation output, which is defined as follows:

$$L_{Dice}=1- DSC=1- \frac{2\left( P\cap T \right)}{\left| P \right|+ \left| T \right|} (Eq. 2)$$

where ∩ is the intersection and *(P* ∩ *T)* represents the spatial overlap between *P* and *T*. *T* denotes the ground truth segmentation and *P* denotes the predicted segmentation. | *P* | and | *T* | represent the areas of *P* and T, respectively.

Training was conducted using Python (v3.8.10) and nnU-Net (v2.0) on NVIDIA GPUs, including Quadro RTX 8000, A100, and H100. The entire experiment was conducted using Python (v3.8.10)^11^ and nnU-Net (v2.0)^12^ using various NVIDIA GPUs, including Quadro RTX 8000, A100 and H100.^13^

Validation

To reinforce the robustness of our segmentation network, we further substantiated our results by evaluating the network’s performance across two different validation datasets.

1. Internal Validation Set: Data from the Validation Set 1 consisted from Site 2 that facilitated the reproducibility of our results.
2. External Validation Set: Data from the Validation Set 2 came from an independent institution, Site 3, which had not contributed prior datasets to the initial training. This dataset enabled a more extensive validation of our network's generalizability and accuracy, independent of the specific local imaging protocols and CT scanner manufacturers.

Statistical Analyses

#### Segmentation

The performance of each network for the segmentation estimations of ICH, PHE, and IVH was evaluated on test data after the training and validation process as well as in the validation sets. To quantify the quality of the segmentation estimations, we calculated the DSC as an established validation metric of spatial overlap index.^10^ The DSC measured the spatial overlap between two segmentations, the predicted region (P) and the verified ground truth (T) region as elaborated in Equation 2. A paired samples t-test was used to compare the DSC values between the reference standard (repeated ground truth segmentations) and the nnU-Net’s predictions on the validation sets.

*Volume Estimation*

To assess the precision of volume estimations, the AVE was computed to reflect the deviation of predicted volumes from the ground truth as well as RVD, as explicated in Equation 3 and 4.^14,15^ The RVD is the absolute volume difference calculated relative to the volume of the ground truth. Furthermore, we examined the relationship between predicted and ground truth volumes using Pearson's correlation coefficient (R).^14^ The results were reported as point estimates with the corresponding standard deviation, delineated by the double standard deviation from 1000 bootstrap resamples.

$$AVE [ml] =Predicted Volume- Ground Truth Volume (Eq. 3)$$

$$RVD \left( P,T \right)= \frac{Vp-Vt}{\mathrm{Vt}} \left( Eq. 4 \right)$$

Where V_p_ represents the predicted volume and V_t_ the true (ground truth) volume. A perfect score of 0 indicates that the objects have identical volume, but does not necessarily mean that the prostates are well aligned spatially.^15^

*Detection*

To ascertain the performance of our automated detection system, we systematically evaluated its accuracy, sensitivity, and specificity. The latter metrics were calculated by benchmarking the automated detection of ICH, PHE, and IVH against the control group with healthy individuals which comprised N=50 NCCT scans. The mathematical formulations corresponding to the specified metrics are detailed in Equations 5 through 7.

$$Accuracy= \frac{TP+TN}{TP + FN+FP + TN} (Eq. 5)$$

$$Sensitivity= \frac{TP}{TP + FN} (Eq. 6)$$

$$Specificity= \frac{TN}{TN + FP} (Eq. 7)$$

Where TP: True Positive, TN: True Negative, FP: False Positive, FN: False Negative, TP represents the number of samples with a presence of IVH correctly classified as IVH positive, while FN represents the number of IVH samples wrongly classified to negative IVH class. Similarly, TN represents the number of samples without IVH presence correctly classified to as IVH negative cases while FP represents the number of samples without IVH presence wrongly classified as IVH positive. The described method for classification accuracy applies uniformly across other lesion types such as ICH and PHE, where TP, TN, FP, and FN correspondingly indicate correctly or incorrectly classified cases with or without the presence of the respective lesion.

*Subgroup Analysis for ICH and IVH*

The network's capability in identifying lesions for ICH, PHE, and IVH was assessed separately focusing on cases with volumes exceeding 1 ml, as this volume threshold addresses a methodological gap prevalent in both the current literature and FDA-approved hemorrhage detection software.^2,16^ This assessment was also extended to IVH segmentation evaluations as this particular volume threshold has been recognized as a marker of clinical relevance.

A second subgroup analysis was performed to evaluate the network’s accuracy in segmenting ICH when concurrent intra- and extracranial bleedings, such as SAH, SDH, or EDH, were present. This involved comparing DSC for ICH lesions both with and without accompanying hemorrhages, and assessing the correlation between bleeding type and segmentation accuracy using Pearson's correlation coefficient.

A Web-based User-Interface for Radiological Report

The final step involved the development of a web-based user-interface (UI), the ICH-Viewer, for visualization of segmentations and reporting of volumes. The front-end UI was written using the Cornerstone.js - JavaScript library.^17^ Cornerstone is used by default for image retrieval, decoding, and rendering of DICOM images.^18^ It can leverage CPU- and GPU-based rendering to display medical imaging data sets. These services are orchestrated through a back-end realized using Python scripts. In our implementation, our UI is created with Flask, a Python based front end development tool that updates hypertext modeling language (HTML) files.^19^ Information is captured through an HTML form supplemented with JavaScript, which is the processed through a decision support module to provide predictions for the different volumes and presence of IVH. The results are then presented in a web-friendly format which can be copied and pasted into a radiologist’s reporting software. The application includes a robust image viewer alongside the display of segmentation maps created with the network alongside the generated report including volume quantification of ICH, PHE, IVH as well as information on the presence of IVH. Supplementary Figure 2 shows the UI with a representative snapshot of the ICH-Viewer Interface.

**Supplementary Table 1:** Technical Specifications of CT Scanners.

| **CT Scanner Model** | **Manufacturer** | **Number of Detectors** | **Rotation Time [s]** | **KVP [KV]** | **I [mA]** | **FOV [mm]** | **Slice Thickness [mm]** |
| --- | --- | --- | --- | --- | --- | --- | --- |
| **Site 1, Germany** | | | | | | | |
| Aquillion PRIME 160 | Canon Medical Systems Corporate | 160 | 0.35 | 120 | 270 | 500 | 0.5-5 |
| Aquillion PRIME 160 |  | 160 | 0.35 | 120 | 250 | 500 | 0.5-5 |
| Aquilion PRIME 160 |  | 160 | 0.35 | 120 | 280 | 500 | 0.5-5 |
| Aquilion ONE ViSION |  | 320 | 0.28 | 120 | 250 | 500 | 0.5-5 |
| Aquilion PRIME 160 Fluoroscopy |  | 160 | 0.35-0.5 | 120 | 270 | 500 | 0.5-5 |
| Aquilion PRIME 160 |  | 160 | 0.35 | 120 | 180 | 500 | 0.5-5 |
| LightSpeed VCT Ultra 64 | GE Healthcare | 64 | 0.35 | 120 | 100-450 | 500 | 0.625-5 |
| Revolution EVO |  | 64 | 0.28 | 120 | 100-365 | 500 | 0.5-5 |
| Revolution GSI |  | 64 | 0.28 | 120 | 100-365 | 500 | 0.5-5 |
| Revolution CT |  | 256 | 0.28 | 120 | 200-400 | 500 | 0.5-5 |
| **Site 2, Germany** | | | | | | | |
| iCT 256 | Philips Healthcare | 256 | 0.27 | 120 | 350 | 500 | 0.5-5 |
| **Site 3, Germany** |  |  |  | 120 |  |  |  |
| SOMATOM Definition Flash | Siemens Germany Healthcare | 128 | 0.28 | 120 | 300 | 500 | 0.5-5 |
| **Site 4, Italy** | | | | | | | |
| Aquilion PRIME | Canon Medical Systems Corporate | 160 | 0.35 | 120 | 250 | 500 | 0.5-5 |

*Legend:* Key specifications for each CT scanner model by site, per scanner. Columns include "Number of Detectors," "Rotation Time (s)," "KVP [KV]" (kilovoltage), "I [mA]" (milliamperes), "FOV [mm]" (Field of View in millimeters), and "Slice Thickness [mm]." Each row lists specifications for a specific scanner at a given site location.

**Supplementary Table 2:** Scan Distribution by CT Scanner and Location for Site 1.

| **CT Scanner Model** | **Manufacturer** | **Location** | **Total Scans** | **Percentage of Total Scans (%)** | **Manufacturer Share (%)** |
| --- | --- | --- | --- | --- | --- |
| Aquillion PRIME 160 | Canon Medical Systems Corporate | Campus 1 | 186 | 23.13 | 55.46 |
| Aquillion PRIME 160 |  |  | 1 | 0.12 |  |
| Aquilion PRIME 160 |  |  | 33 | 4.1 |  |
| Aquilion ONE ViSION |  |  | 5 | 0.62 |  |
| Aquilion PRIME 160 Fluoroscopy | Canon Medical Systems Corporate | Campus 2 | 133 | 16.54 |  |
| Aquilion PRIME 160 |  |  | 88 | 10.95 |  |
| LightSpeed VCT Ultra 64 | GE Healthcare | Campus 3 | 183 | 22.76 | 44.53 |
| Revolution EVO |  |  | 36 | 4.48 |  |
| Revolution GSI |  |  | 36 | 4.48 |  |
| Revolution CT |  |  | 103 | 12.81 |  |

*Legend:* CT scanner models by manufacturer and location for **site 1**, with scan counts and percentages. "CT Scanner Model" lists each model, "Manufacturer" specifies the company, and "Location" indicates the campus. "Total Scans" is the number of scans per scanner, with "Percentage of Total Scans (%)" showing each model's share of the total 804 scans. "Manufacturer Share (%)" gives the cumulative scan percentage for each manufacturer.

**Supplementary Table 3:** Patient and Imaging Characteristics Across Training, Test, and Validation Sets.

| **Characteristics** | **Training**  **Set**  **(N= 775)** | **Test**  **Set**  **(N= 189)** | **Validation**  **Set 1**  **(N= 121)** | **Validation**  **Set 2**  **(N= 169)** |
| --- | --- | --- | --- | --- |
| Age [years], median (IQR) | 73 (60-79) | 73 (63-81) | 69 (75-80) | 69 (55-79) |
| T-Test vs training set; p-value |  | 0.115 | 0.329 | 0.025 |
| Sex [female], n (%) | 325 (41.9) | 97 (51.3%) | 45 (37.5) | 92 (53.5) |
| T-Test vs training set; p-value |  | 0.102 | 0.372 | 0.440 |
| Systolic Blood Pressure [mmHg], median (IQR) | 170  (144.5-200) | 163.50 (142-190) | 159  (132-188) | 196 (178.75-242.5) |
| T-Test vs training set; p-value |  | 0.192 | 0.002 | <0.001 |
| History of hypertension, n (%) | 606 (78.2) | 156 (82.5) | 80 (66.1) | 92 (53.5) |
| T-Test vs training set; p-value |  | 0,162 | 0.007 | <0.001 |
| Oral anticoagulation, n (%) | 209 (27) | 47 (24.9) | 30 (24.8) | 35 (20.3) |
| T-Test vs training set; p-value |  | 0.582 | 0.740 | 0.120 |
| Antiplatelet medication, n (%) | 355 (45.8) | 92 (48.7%) | 57 (47.1) | 59 (34.4) |
| T-Test vs training set; p-value |  | 0.515 | 0.768 | 0.013 |
| GCS at baseline, median (IQR) | 13 (7-15) | 12 (6.5;15) | 13 (6-15) | 12 (6;13) |
| T-Test vs training set; p-value |  | 0.740 | 0.857 | 0.002 |
| symptom onset to imaging [hours], median (IQR) | 4.5  (1.81-15.85) | 3.92 (1.48-11.04) | 2.2  (1.28-4.74) | NA |
| T-Test vs training set; p-value |  | 0.129 | <0.001 | NA |
| mRS 4-6, n (%) | 574 (73.7) | 148 (78.3) | 84 (69.4) | 100 (58.1) |
| T-Test vs training set; p-value |  | 0.501 | 0.172 | <0.001 |
| Missing, n (%) | 20 (3.1) | 1 (0.5) | 1 (0.8) | 4 (2.3) |
| ICH location |  |  |  |  |
| Lobar, n (%) | 342 (44.1) | 80 (42.3) | 56 (46.3) | 75 (43.6) |
| T-Test vs training set; p-value |  | 0.683 | 0.622 | 0.932 |
| Deep, n (%) | 313 (40.4) | 80 (42.3) | 54 (44.6) | 85 (49.4) |
| T-Test vs training set; p-value |  | 0.618 | 0.365 | 0.024 |
| Brainstem/Pons, n (%) | 40 (5.2) | 9 (4.8) | 1 (0.8) | 5 (2.9) |
| T-Test vs training set; p-value |  | 0.885 | 0.001 | 0.014 |
| Cerebellum, n (%) | 80 (10.3) | 20 (10.6) | 10 (8.3) | 7 (4.1) |
| T-Test vs training set; p-value |  | 0.873 | 0.569 | 0.226 |
| ICH Vol [mL], median (IQR) | 22.95 (8.31-52.75) | 21 (5.93-  42.09) | 24.53 (7.8-56.46) | 28.57 (11.14-55.39) |
| T-Test vs training set; p-value |  | 0.145 | 0.735 | 0.209 |
| IVH presence, n (%) | 380 (49) | 90 (47.6) | 59 (49.17) | 59 (35.12) |
| T-Test vs training set; p-value |  | 0.746 | <0.001 | <0.001 |
| IVH Vol [mL], median (IQR) | 7.98 (2.66-19.77) | 8.31 (2.48-19.74) | 12.1 (3.24-31.64) | 12.49 (7.04-23.27) |
| T-Test vs training set; p-value |  | <0.001 | <0.001 | <0.001 |
| PHE Vol [mL], median (IQR) | 20.66 (9.36-42.8) | 20.48  8.01-41.54 | 16.2  6.63-34.97 | 32.38  13.89-60.13 |
| T-Test vs training set; p-value |  | 0.315 | 0.003 | 0.002 |

*Legend:* GCS, Glasgow Coma Scale; ICH, intracerebral hemorrhage; IQR, interquartile range; IVH, intraventricular hemorrhage; mRS, modified Rankin Scale; PHE, perihematomal edema; SD, standard deviation; Vol, volume. The volumes reported for ICH, PHE, and IVH are derived from ground truth segmentations.

**Supplementary Table 4:** Sensitivity and Specificity for IVH Detection across different Datasets.

|  | **Treshhold of 0.2 ml** | |
| --- | --- | --- |
| **Data Set** | **Sensitivity** | **Specificity** |
| Test Set | 0.94 (0.88-0.99) | 0.91 (0.85-0.96) |
| Validation Set 1 | 0.88 (0.82-0.94) | 0.96 (0.92-1) |
| Validation Set 2 | 0.73 (0.64-0.81) | 0.91 (0.83-0.99) |

**Legend:** Sensitivity and specificity rates for IVH detection plotted for volumes above 0.2 mL across various datasets

**Supplementary Table 5:** Comparative Analysis of Segmentation Precision in Parenchymal Hemorrhages with other coexisting Intracranial Hemorrhage Types.

| **Presence of SAH** | **Yes** | **No** | **p-value** |
| --- | --- | --- | --- |
| Mean DICE Score | 0.90 ± 0.04 | 0.86 ± 0.14 | 0.3067 |
| Median DICE Score | 0.90 ± 0.05 | 0.91 ± 0.07 |  |
| Pearson Correlation Coefficient | 0.075 | |  |
|  |  |  |  |
| **Presence of SDH and/or EDH** | **Yes** | **No** | **p-value** |
| Mean DICE Score | 0.87 ± 0.05 | 0.87 ± 0.14 | 0.71858 |
| Median DICE Score | 0.91 ± 0.06 | 0.90 ± 0.07 |  |
| Pearson Correlation Coefficient | 0.027 | |  |

*Legend:* Mean and Median Dice Similarity Coefficients for Segmentation of Intracerebral Hemorrhage with or without Extension into Subarachnoid, Epidural, or Subdural Spaces, Including Associated Correlation Coefficients. DICE, Dice Similarity Coefficients; EDH, epidural hematoma; SAH, subarachnoid hemorrhage; SDH, subdural hematoma.

**Supplementary Figure 1:** Deep Learning Architecture of the customized 3D U-Net in the nnU-Net.


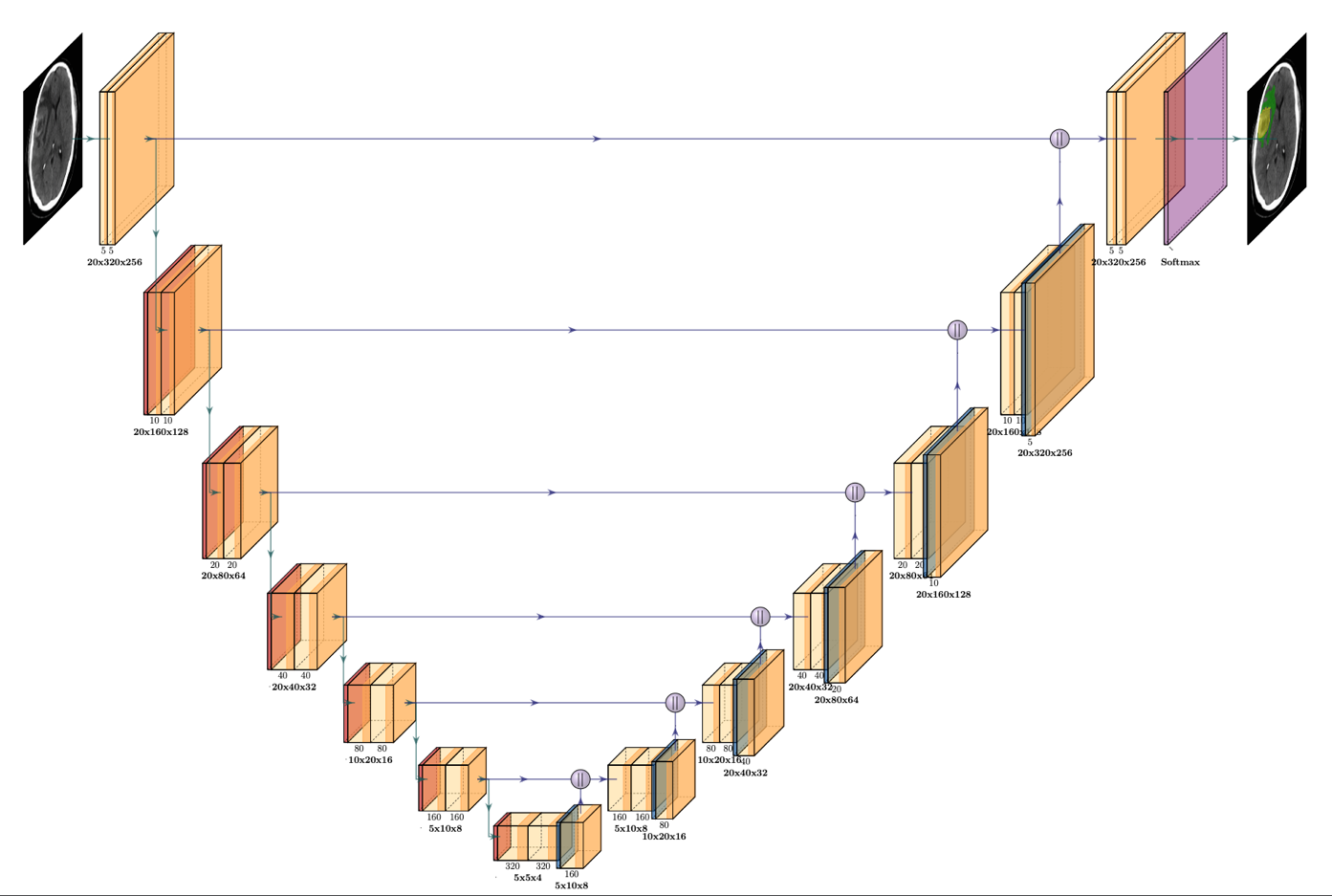


*Legend:* Illustrated nnU-Net framework for automated segmentation of intracerebral hemorrhage (ICH), perihematomal edema (PHE), and intraventricular hemorrhage (IVH) involving several steps: **(1) Data Preprocessing:** The initial stage involves preprocessing of input data, which includes standardizing intensity and resolution to prepare for segmentation. **(2)** **Segmentation Mechanism:** Utilizing nnU-Net architecture, the algorithm employs a block that contains a 3x3x3 convolution, instance normalization, and ReLU activations for segmenting ICH, PHE, and IVH. **(3)** **Volumetric Quantification:** Following segmentation, the volumes of ICH, IVH, and PHE are calculated. **(4) Network Configuration and Training:** nnU-Net fully automatically configures and trains the entire segmentation pipeline, tailored to the dataset's characteristics. (5) Adaptive Scaling: The network can adjust to the input patch size, allowing up to seven down-sampling and up-sampling operations, potentially increasing the feature representation significantly at the bottleneck layer. **(6)** **Softmax Function:** Finally, a softmax function is applied to the output layers to produce probabilistic maps, which assist in distinguishing between the different types of hemorrhagic lesions.

**Supplementary Figure 2:** ICH-Viewer Interface: Integrative Display for Image Analysis and Volume Reporting.


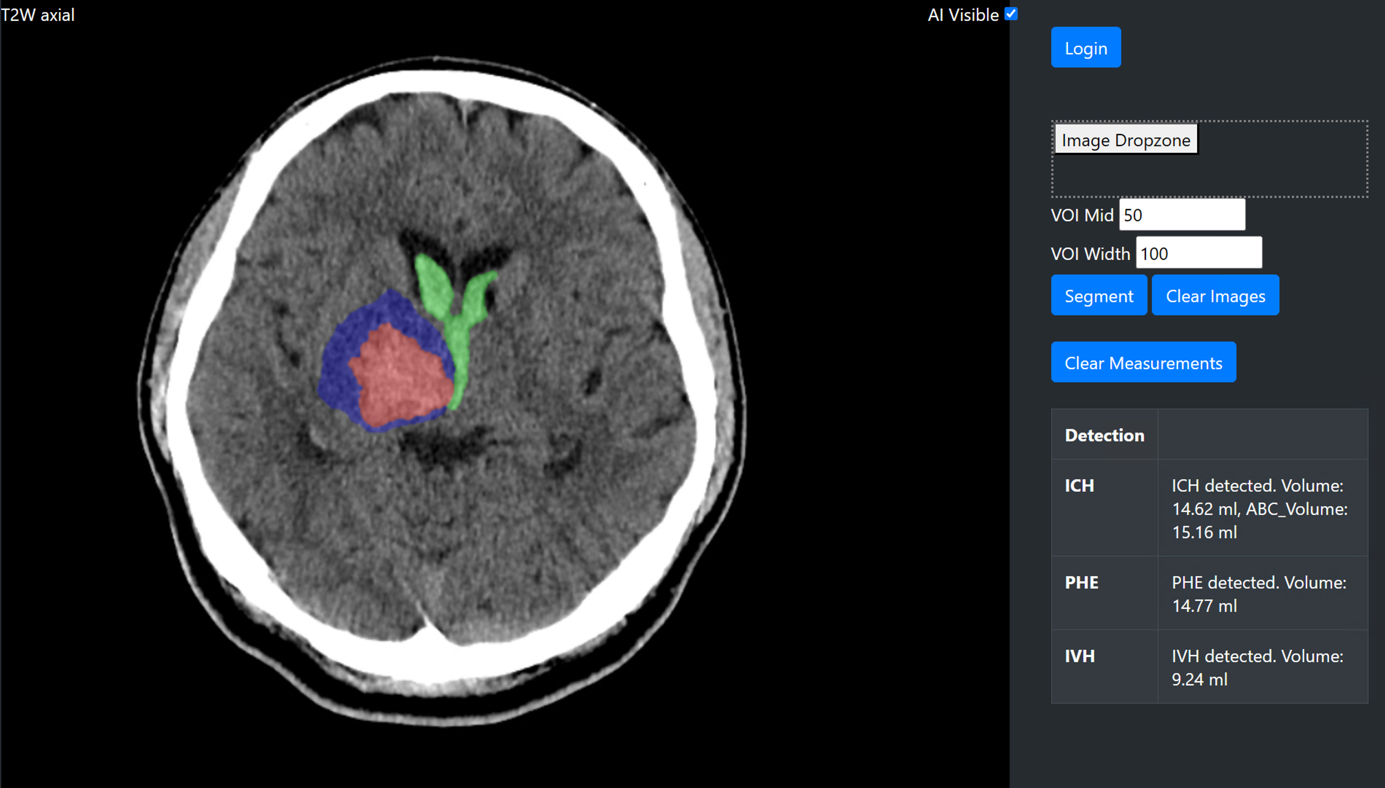


*Legend:* Interface Snapshot: Automated segmentation visualization for intracerebral hemorrhage (red) in the basal ganglia with adjacent perihematomal edema (blue) and intraventricular involvement (green). Side panels feature a DICOM or Nifti dropzone for ordering or clearing the segmentation and a dedicated viewport displaying automated volumetric reports alongside volumes generated via the ABC/2 Method for ICH assessment. ICH, intracerebral hemorrhage; IVH, intraventricular hemorrhage; PHE, perihematomal hemorrhage.

**Supplementary Figure 3**: Correlation Scatter Plot Analysis for Performance on Test Set and Validation Sets.

*Legend:* Scatter plots illustrating the correlation between automated segmentation volumes and ground truth across various data sets. Each point represents the automated volume versus the manual (ground truth) volume for intracerebral hemorrhage (ICH), perihematomal edema (PHE), and intraventricular hemorrhage (IVH). Panel A focuses on the test set, while Panels B and C depict validation sets for spontaneous ICH cases—internal and external, respectively. Panel D examines an internal validation set covering a range of different ICH etiologies. The trend lines indicate the direction and strength of the correlation, providing insight into the predictive accuracy of the segmentation model. The shaded blue region around the trend line in the scatter plot

**Supplementary Figure 4:** Volume-dependent Variances in Sensitivity and Specificity for IVH Detection.

*Legend:* This figure illustrates the relationship between IVH volume and the sensitivity (blue) and specificity (orange) of IVH detection across four datasets: the test set (A) and the two validation sets (B and C). The graphs display how sensitivity and specificity rates vary with increasing IVH volume thresholds, indicating the model's optimal metrics at between 0.2-0.25 ml.

**Supplementary Figure 5:** Segmentation Performance for ICH with Associated Intra- and Extraaxial Bleedings.


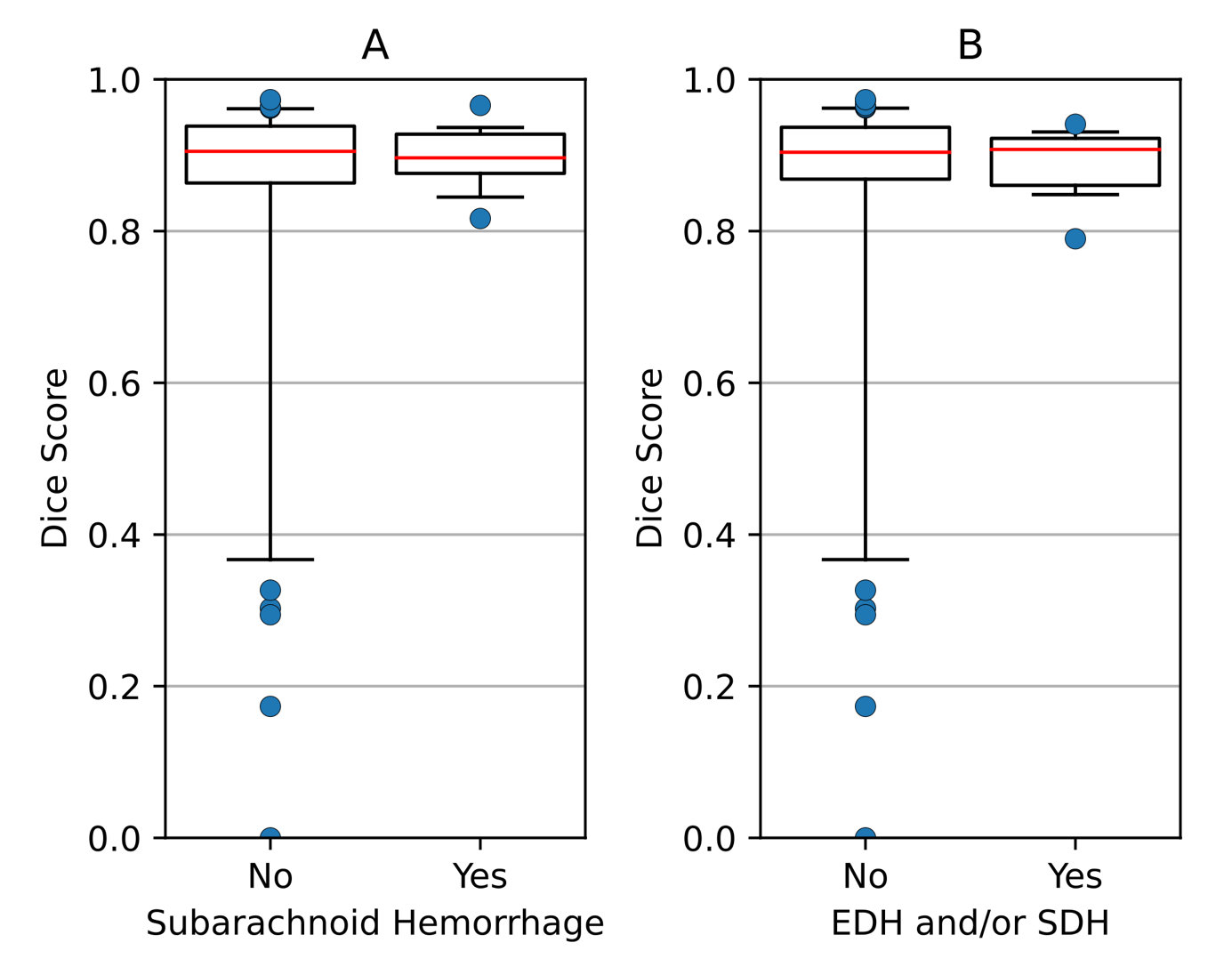
*Legend:* Series of boxplots illustrating the comparative accuracy of the network's segmentation predictions for cases of intracerebral hemorrhage (ICH) with concurrent intra- or extraaxial bleedings. A: Dice similarity coefficient (DSC) for segmentations when subarachnoid hemorrhage is present. B: DSC for cases with either epidural (EDH) or subdural hematoma (SDH). Each boxplot represents the distribution of Dice scores, with the central line indicating the median, the box denoting the interquartile range (IQR), and the whiskers extending to the most extreme data points that are no more than 1.5 times the IQR from the upper and lower quartiles. Outliers are not individually plotted to preserve the clarity of the visualization.

**Supplementary Figure 6:** Comparative Analysis of Segmentation Accuracy Across Test and different Validation Sets for ICH, PHE, and IVH Labels.

**Legend:** Boxplot comparison of DICE Scores for segmentation accuracy across different sets. Panel A presents mean DICE Scores, while Panel B shows median values. The metrics are compared for intracerebral hemorrhage (ICH), perihematomal edema (PHE), and intraventricular hemorrhage (IVH). Color coding represents the test set (blue), validation sets (internal validation in red, external validation in green). DICE scores from repeated manual segmentations are defined as the reference standard (orange). Whiskers extend to the most extreme data points within 1.5 times the 95% CI from the quartiles, and outliers are omitted from individual plotting to maintain visual clarity.

**Supplementary Figure 7:** Evaluation of Imaging Artifacts - A Visual Comparison of Model Accuracy.

*Legend:* Noncontrast Computed Tomography scans with the original data (left column), ground truth segmentations (mid column) and the network’s predicted segmentations (nnU-Net, right column). A: High accuracy in identifying all lesions in case with beam hardening artifact B: Moderate accuracy in identifying all lesions in case with motion artifact. The color-coding is as follows: intracerebral hemorrhage (ICH) is highlighted in red; and perihematomal edema (PHE) is depicted in blue.

# Supplementary References

1. Nawabi J, Kniep H, Elsayed S, Friedrich C, Sporns P, Rusche T, et al. Imaging-Based Outcome Prediction of Acute Intracerebral Hemorrhage. *Transl. Stroke Res. 2021* [Internet]. 2021;1–10. A

2. Nawabi J, Schlunk · Frieder, Orco AD, Elsayed S, Federico Mazzacane ·, Desser D, et al. Non-contrast computed tomography features predict intraventricular hemorrhage growth. *Eur. Radiol. 2023* [Internet]. 2023;1:1–11.

3. Gao R, Khan MS, Tang Y, Xu K, Deppen S, Huo Y, et al. Technical Report: Quality Assessment Tool for Machine Learning with Clinical CT. 2021 [cited 2023 Nov 22];Available from: https://arxiv.org/abs/2107.12842v1

4. Vogt E, Vu LH, Cao H, Speth A, Desser D, Schlunk F, et al. Multilesion Segmentations in Patients with Intracerebral Hemorrhage: Reliability of ICH, IVH and PHE Masks. *Tomogr. (Ann Arbor, Mich.)*. 2023;9:89–97.

5. Yushkevich PA, Piven J, Hazlett HC, Smith RG, Ho S, Gee JC, et al. User-guided 3D active contour segmentation of anatomical structures: Significantly improved efficiency and reliability. *Neuroimage*. 2006;31:1116–1128.

6. Isensee F, Jaeger PF, Kohl SAA, Petersen J, Maier-Hein KH. nnU-Net: a self-configuring method for deep learning-based biomedical image segmentation. *Nat. Methods 2020 182*. 2020;18:203–211.

7. Isensee F, Jager PF, Kohl SAA, Petersen J, Maier-Hein K. Automated Design of Deep Learning Methods for Biomedical Image Segmentation. *arXiv Comput. Vis. Pattern Recognit.* 2019;

8. Zhao X, Chen K, Wu G, Zhang G, Zhou X, Lv C, et al. Deep learning shows good reliability for automatic segmentation and volume measurement of brain hemorrhage, intraventricular extension, and peripheral edema. *Eur. Radiol*. 2021;31:5012–5020.

9. Mao A, Mohri M, Zhong Y. Cross-Entropy Loss Functions: Theoretical Analysis and Applications. *Proc. Mach. Learn. Res*. 2023;202:23803–23828.

10. Zou KH, Warfield SK, Bharatha A, Tempany CMC, Kaus MR, Haker SJ, et al. Statistical Validation of Image Segmentation Quality Based on a Spatial Overlap Index: Scientific Reports. *Acad. Radiol.*. 2004;11:178.

11. Python Release Python 3.8.10 | Python.org [Internet]. [cited 2024 Jan 7];Available from: https://www.python.org/downloads/release/python-3810/

12. GitHub - MIC-DKFZ/nnUNet [Internet]. [cited 2022 Dec 19];Available from: https://github.com/MIC-DKFZ/nnUNet

13. NVIDIA® Virtual GPU Software Supported GPUs [Internet]. [cited 2024 Jan 7];Available from: https://docs.nvidia.com/grid/gpus-supported-by-vgpu.html

14. Wendler T, Kreissl MC, Schemmer B, Rogasch JMM, De Benetti F. Artificial Intelligence-powered automatic volume calculation in medical images - available tools, performance and challenges for nuclear medicine. *Nuklearmedizin*. 2023;62:343–353.

15. Isaksson LJ, Pepa M, Summers P, Zaffaroni M, Vincini MG, Corrao G, et al. Comparison of automated segmentation techniques for magnetic resonance images of the prostate. *BMC Med. Imaging* [Internet]. 2023;23:1–16.

16. Sreekrishnan A, Venkatasubramanian C, Heit JJ. Automated cerebral hemorrhage volume calculation and stability detection using automated software. *Res. Sq.* [Internet]. 2023 [cited 2023 Nov 22];Available from: https://pubmed.ncbi.nlm.nih.gov/37292654/

17. Cornerstone.js | Cornerstone.js [Internet]. [cited 2024 Jan 5];Available from: https://www.cornerstonejs.org/

18. Ziegler E, Urban T, Brown D, Petts J, Pieper SD, Lewis R, et al. Open Health Imaging Foundation Viewer: An Extensible Open-Source Framework for Building Web-Based Imaging Applications to Support Cancer Research. *JCO Clin. Cancer Informatics*. 2020;4:336–345.

19. Welcome to Flask — Flask Documentation (3.0.x) [Internet]. [cited 2024 Jan 5];Available from: https://flask.palletsprojects.com/en/3.0.x/
